# Supplementary material for: Willingness to work in rural areas and associated factors among graduating health students at the University of Gondar, northwest Ethiopia, 2021
Source: PLoS One. 2022 Oct 21;17(10):e0276594. doi: 10.1371/journal.pone.0276594 (PMC9586395; doi:10.1371/journal.pone.0276594)
Supplement: S1 Questionnaire — (DOCX) [file pone.0276594.s001.docx]

English and Amharic version questionnaire

Part I: Socio-demographic and health status characteristics

| Sr. No | Question | | | Answer | | | | | | |  |  |
| --- | --- | --- | --- | --- | --- | --- | --- | --- | --- | --- | --- | --- |
| 101 | How old are you? | | | _____________in years | | | | | | |  |  |
| 102 | Your sex? | | | 1. Male 2. Female | | | | | | |  |  |
| 103 | What’s your department? | | |  | | | | | | |  |  |
| 104 | What’s your place of birth | | | 1. Urban 2 sub-urban 3 Rural | | | | | | |  |  |
| 105 | Current living location ( permanent residency out of campus) | | | 1. Urban 2. Sub urban 3. Rural | | | | | | |  |  |
| 106 | What is your marital status | | | 1.single  2. Married 3.In a relationship | | | | | | |  |  |
| 107 | How much is your monthly pocket money? | | | ____________in birrs | | | | | | |  |  |
| 108 | Do you have a smart phone or computer? | | | 1. Yes 2. No | | | | | | |  |  |
| 109 | What is your religion? | | | 1. Orthodox 3. Protestant 2. Muslim 4.other | | | | | | |  |  |
| 110 | What is your mother’s educational status? | | | 1 have no formal education   1. primary education(1-8) 2. secondary education(9-12) 3. collage and above | | | | | | |  |  |
| 111 | What is your father’s educational status? | | | 1.have no formal education  2.primary education(1-8)  3.secondary education(9-12)  4.collage and above | | | | | | |  |  |
| 112 | What is your mother’s occupation? | | | 1.Farmer  2.Merchant  3.Government employee  4.Private employee  5.Others please specify____________ | | | | | | |  |  |
| 113 | What is your father’s occupation? | | | 1.Farmer  2.Merchant  3.Government employee  4.Private employee  5.Others please specify____________ | | | | | | |  |  |
| 114 | What is the average monthly income of the family? | | | _________________________ | | | | | | |  |  |
| 115 | Have you ever chewed chat? | | | 1 yes 2. No | | | | | | |  |  |
| 116 | If yes, how often? | | | 1. Everyday 2. once per week 3. twice per week 4. three or more times per week | | | | | | |  |  |
| 117 | Have you ever smoked cigarette? | | | 1. Yes 2. No | | | | | | |  |  |
| 118 | If yes how often? | | | 1. Everyday 2. once per week 3. twice per week   4.three or more times per week | | | | | | |  |  |
| 119 | Have you ever drunk alcohol? | | | 1. Yes 2 no | | | | | | |  |  |
| 120 | If yes, how often? | | | 1.Everyday  2. once per week  3.twice per week  4.three or more times per week | | | | | | |  |  |
| 121 | Do you have a known medical problem? | | | 1. Yes 2. No | | | | | | |  |  |
| 122 | If yes, what medical problem did you have? | | ___________________________ | | | | | | | |  |  |
| 123  123 | Have you ever seen a mother die because of lack of healthcare provider? | | 1. Yes 2. No | | | | | | | |  |  |
| Academic and career related characteristics | | | | | | | | | | |  |  |
| 201 | Did you have any information about the department you joined before campus entry? | 1. Yes 2. No | | | | | | | | |  |  |
| 202 | If yes, what was your source of information? | 1. Healthcare providers 2. Family 3. Mass media 4. Friends 5. if others please specify____________ | | | | | | | | |  |  |
| 203 | Was the department you joined your first choice? | 1. Yes 2. No | | | | | | | | |  |  |
| 204 | If no, what was your feeling when you heard about your department the first time? | 1. I was happy 2. I was sad.  3. I had no idea | | | | | | | | |  |  |
| 205 | What is your CGPA | _______________ | | | | | | | | |  |  |
| 206 | Do you have an intention to continue with your current department or profession? | 1 yes 2. No | | | | | | | | |  |  |
| 207 | Where do you think jobs are more available in Ethiopia? | 1. Urban areas 2. Rural areas | | | | | | | | |  |  |
| 208 | After graduation, you will have chance to be assigned in rural areas, are you willing to work in the rural settings? | Yes 2. No | | | | | | | | |  |  |
| 209 | If no, for question number 33 write down all your perceived reasons on the space provided (in Amharic or English |  | | | | | | | | |  |  |
| 36.Attitude related questions | | | | | | | | | | | | 5=strongly agree |
| SR.N | Questions | | 5=Strongly agree | | | 4=agree | | 3=neutral | 2=disagree | 1=strongly disagree |  |  |
| A | Working in these areas provides opportunities to use various skills | |  | | |  | |  |  |  |  |  |
| B | There are supportive environment when working in these environment | |  | | |  | |  |  |  |  |  |
| C | Working in these areas limits communication with professional peers | |  | | |  | |  |  |  |  |  |
| D | Working in these areas provides opportunities to work independently | |  | |  | |  | |  |  |  |  |
| E | There are lack of amenities and entertainment in these areas | |  | |  | |  | |  |  |  |  |
| F | People in these areas are friendly | |  | |  | |  | |  |  |  |  |
| G | Working in these areas causes isolation from family and friends | |  | |  | |  | |  |  |  |  |
| H | Working as health care provider in hospitals or health centers in these areas is the most important  contribution to the health of the population | |  | |  | |  | |  |  |  |  |
| I | Health science collage prepared me well to work in these areas | | |  |  | |  | |  |  |  |  |
| J | Working in hospitals these areas is the most challenging | | |  |  | |  | |  |  |  |  |
| K | Working in the hospital in these areas provides opportunities for real life problem solving | | |  | |  | |  |  |  |  |  |

# Annex II Amharic version questionnaire

**ክፍል አንድ: ማህበራዊ እና ኢኮኖሚያዊ ነክ ጥያቄዎች**

| ቁ | መጠይቅ | የተሰጡ አማራጮች | ኮድ | ይዝለሉ | |  |
| --- | --- | --- | --- | --- | --- | --- |
| 101 | እድሜዎ ስንት ነው | ________________ |  |  | |  |
| 102 | የእርስዎ ጾታ? | ሀ/ወንድ ለ/ሴት |  |  | |  |
| 103 | የእርስዎ የትምህርት ክፍል ምንድነው? |  |  |  | |  |
| 104 | የትውልድ ቦታዎ የትነው? | ሀ/የከተማ ለ/ክፍለ ከተማ ሐ/ገጠር |  |  | |  |
| 105 | የጋብቻ ሁኔታዎ ምንድነው | ሀ/ ያላገባች  ለ/ ያገባ  ሐ/በእጮኝነት ያለ |  |  | |  |
| 106 | በየወሩ የኪስ ገንዘብዎ ስንት ነው? | ______በብር |  |  | |  |
| 107 | ዘመናዊ ስልክ ወይም ኮምፒተር አለዎት? | ሀ/አዎ ለ/ አይደለም |  |  | |  |
| 108 | ሃይማኖትዎ ምንድን ነው? | ሀ/ኦርቶዶክስ ሐ/ፕሮቴስታንት  ለ/ሙስሊም መ /ሌላ |  | | |  |
| 109 | የእናትዎ የትምህርት ደረጃ | ሀ/ መደበኛ ትምህርት የላትም  ለ/የመጀመሪያ ደረጃ ትምህርት (1-8)  ሐ/ሁለተኛ ደረጃ ትምህርት (9-12)  መ/ኮሌጅ ​​እና ከዚያ በላይ |  | | |  |
| 11ዐ | የእናትዎ ሥራ ምንድነው? | ሀ/አርሶ አደር /የቤት እመቤት  ለ/ ነጋዴ  ሐ/የመንግስት ሰራተኛ  መ/ የግል ሠራተኛ  ሠ/ሌላ ከሆነ እባክዎን ይግለጹ____________ |  | | |  |
| 110 | የአባትዎ የትምህርት ደረጃ | ሀ/ መደበኛ ትምህርት የለውም  ለ/የመጀመሪያ ደረጃ ትምህርት (1-8)  ሐ/የሁለተኛ ደረጃ ትምህርት (9-12)  መ/ ኮሌጅ እና ከዚያ በላይ |  | | |  |
| 111 | የአባትዎ ሥራ ምንድነው? | ሀ/አርሶ አደር  ለ/ ነጋዴ  ሐ/ የመንግስት ሰራተኛ  መ/ የግል ሠራተኛ  ሠ/ ሌላ ከሆነ እባክዎን ይግለጹ___________ |  | |  |  |
|  |  |  |  | | |  |
| 112 | የቤተሰቡ አማካይ ወርሃዊ ገቢ ስንትነው? | _________በብር |  | | |  |
| 113 | ጫት ቅመው ያውቃሉ? | ሀ/ አዎ ለ/የለም | አዎ ከሆነ ወደ ጥያቄ 114 | | |  |
| 114 | አዎ ከሆነ ፣ ስንት ጊዜ? | ሀ/በየቀኑ  ለ/በሳምንት አንድ ጊዜ  ሐ/በሳምንት ሁለት ጊዜ  መ/በሳምንት ሦስት ወይም ከዚያ በላይ ጊዜ |  | | |  |
| 115 | .ሲጋራ አጭሰው ያውቃሉ? | ሀ/አዎ ለ/የለም | አዎ ከሆነ ወደ ጥያቄ 116 | | |  |
| 116 | አዎ ከሆነ ስንት ጊዜ? | ሀ/በየቀኑ  ለ/በሳምንት አንድ ጊዜ  ሐ/በሳምንት ሁለት ጊዜ  መ/በሳምንት ሶስት ወይም ከዚያ በላይ ጊዜ |  | | |  |
| 117 | አልኮል ጠጥተው ያውቃሉ? | ሀ/አዎ ለ/ የለም | አዎ ከሆነ ወደ ጥያቄ 118 | | |  |
| 118 | አዎ ከሆነ ስንት ጊዜ? | ሀ/ በየቀኑ  ለ/በሳምንት አንድ ጊዜ  ሐ/ በሳምንት ሁለት ጊዜ  መ/በሳምንት ሶስት ወይም ከዚያ በላይ ጊዜ |  | | |  |
| 119 | በሕክምና የተረጋገጠ ችግር አለብዎት? | ሀ/አዎ ለ/ የለም |  | | |  |
| 120 | አዎ ከሆነ ምን ዓይነት የሕክምና ችግር ነበረብዎት? | ___________________ |  | | |  |
| 121 | ቴሌቪዥን ተመልክተው ያውቃሉ? | ሀ/አዎ ለ/ የለም | አዎ ከሆነ ወደ ጥያቄ 122 | | |  |
| 122 | አዎ ከሆነበሳምንት ስንት ጊዜ ተመልክተዋል? | ሀ/በየቀኑ  ለ/በሳምንት አንድ ጊዜ  ሐ/በሳምንት ሁለት ጊዜ  መ/በሳምንት ሦስት ወይም ከዚያ በላይ |  | | |  |
| 123 | ጋዜጣ አንብበዉ ያዉቃሉ | 1. አዎ  2. የለም | አዎ ከሆነ ወደ ጥያቄ124 | | |  |
| 124 | አዎ ከሆነ ምን ያክል ጊዜ ያነባሉ | ሀ/በየቀኑ  ለ/በሳምንት 1 ጊዜ  ሐ/በሳምንት 2 ጊዜና ከዚያ በላይ  መ/ ሌላ ካለ ይገለጽ |  | | |  |
| 125 | ሬድዮ አዳምጠዉ ያዉቃሉ | ሀ/አዎ  ለ/. የለም | አዎ ከሆነ ወደ ጥያቄ 126 | | |  |
| 126 | አዎ ከሆነ ምን ያክል ጊዜ ያዳምጣሉ | ሀ/በየቀኑ  ለ/በሳምንት 1 ጊዜ  ሐ/በሳምንት 2 ጊዜና ከዚያ በላይ  መ/ ሌላ ካለ ይገለጽ |  | | |  |
| 123 | .በጤና እንክብካቤ እጥረት ምክንያት እናት ስትሞት አይተው ያውቃሉ? | ሀ/አዎ ለ/ የለም |  | | |  |

**ከትምህርት እና ከሙያ ጋር ተዛማጅ ባህሪዎች**

| 201 | ካምፓስ ከመግባዎ በፊት ስለተቀላቀሉት የትምህርት ክፍል መረጃ ነበርዎት? | ሀ/ አዎ ለ/የለም |  |
| --- | --- | --- | --- |
| 202 | .አዎ ከሆነ የመረጃዎ ምንጭ ምን ነበር? | ሀ/ከጤና እንክብካቤ አቅራቢዎች  ለ/ከቤተሰብ  ሐ/ከመገናኛ ብዙሀን  መ/ከጓደኞች  ሠ/ ከሌላ ከሆነ እባክዎን ይግለጹ____________ |  |
| 203 | አሁን ያሉበት የትምህርት ክፍል የመጀመሪያ ምርጫዎ ነበር? | ሀ/አዎ ለ/ አይደለም | አይደለም ከሆነ ወደ ጥያቄ 204 |
| 204 | የመጀመሪያ ምርጫዎ ካልሆነ መቀላቀልዎን ሲሰሙ ምን ተሰማዎት? | ሀ/ ደስተኛ ነበርኩ ለ/አዘንኩ/ተከፍቻለሁ  ሐ/እኔ ምንም ሀሳብ አልነበረኝም |  |
| 205 | የእርስዎ እስከ አሁን ያለው አማካኝ ውጤት(CGPA) ስንትነው? | _______________ |  |
| 206 | አሁን ባለው የትምህርት ክፍልዎ ወይም በሙያዎ ለመቀጠል ሀሳብ አለዎት? | ሀ/ አዎ ለ/የለኝም |  |
| 207 | ኢትዮጵያ ውስጥ ሥራ በአብዝሃኛው የሚገኝ የት ይመስልሃል? | ሀ/በከተማ አካባቢዎች  ለ/ በገጠር አካባቢዎች |  |
| 208 | ከተመረቁ በኋላ በገጠር አካባቢዎች የመመደብ እድል ቢኖርዎት በገጠር አከባቢዎች ለመስራት ፈቃደኛ ነዎት? | ሀ/አዎ ለ/አይደለሁም | አይደለሁምከሆነ ወደ ጥያቄ 209 |
| 209 | ለጥያቄ ቁጥር 208 አይደለሁም ካሉ ገጠር ለመሥራት ፈቃደኛ ያልሆኑበትን ምክንያት ከዚህ በታች ባለው ክፍት ቦታ እባክዎ ይግለጹ (በአማርኛ ወይም በእንግሊዝኛ) | __________________________________________  ___________________________________________  ___________________________________________  ___________________________________________ |  |
|  | የአዲስ ተመራቂዎችን በገጠር የመሥራት ፈቃደኝነትን (ፍላጎት) ለማሳደግ መንግስት ምን ማድረግ አለበት ብለው ያስባሉ?(በክፍት ቦታው ይጻፉ) | _____________________________________  _____________________________________  ____________________________________  _____________________________________- |  |

**ከአመለካከት ጋር የተዛመዱ ጥያቄዎች**

| 1 |  | 5= -በጣም እስማማለሁ | | 4-እስማማለሁ | | 3-ገለልተኛ | | 2 -አልስማማም | 1 በጣም  አልስማማም |  |
| --- | --- | --- | --- | --- | --- | --- | --- | --- | --- | --- |
| 301 | አካባቢዎች መስራት የተለያዩ ክህሎቶችን ያሳድጋል ብለው ያስባሉ? |  | |  | |  | |  |  |  |
| 302 | አካባቢዎች ውስጥ በሚሠሩበት ጊዜ አካባቢው ድጋፍ ይሰጣል ብለው ያስባሉ? |  | |  | |  | |  |  |  |
| 303 | አካባቢዎች መሥራት ከሙያ እኩዮች ጋር መግባባትን ይገድባል ብለው ያስባሉ? |  | |  | |  | |  |  |  |
| 304 | አካባቢዎች ውስጥ መሥራት በተናጥል ለመስራት አቅምን ይጨምራል ብለው ያስባሉ?( |  | |  | |  | |  |  |  |
| 305 | አካባቢዎች የመዝናኛ እጥረት አለ ብለው ያስባሉ? | |  |  |  | |  | |  | |
| 306 | አካባቢዎች ያሉ ሰዎች ተግባቢ ናቸው ብለው ያስባሉ | |  |  |  | |  | |  | |
| 307 | አካባቢዎች መሥራት ከቤተሰብ እና ከጓደኞች መነጠልን (መራቅን) ያመጣል ብለው ያስባሉ? | |  |  |  | |  | |  | |
| 308 | በእነዚህ አካባቢዎች በሆስፒታሎች ወይም በጤና ጣቢያዎች ውስጥ የጤና ባለሙያ ሆኖ መሥራት ለህብረተሰቡ ጤና ጉልህ አስተዋጽኦ አለው ብለው ያስባሉ? | |  |  |  | |  | |  | |
| 309 | የተማሩበት ኮሌጅ (ዩኒቨርሲቲ) በእነዚህ አካባቢዎች ለመሥራት በደንብ አዘጋጅቶኛል ብለው ያስባሉ? | |  |  |  | |  | |  | |
| 310 | አካባቢዎች ውስጥ በሚገኙ የጤና ተቋሞች ተቀጥሮ መሥራት ፈታኝ ነው ብለው ያስባሉ | |  |  |  | |  | |  | |
| 311 | አካባቢዎች በሚገኙ የጤና ተቋሞች ውስጥ መሥራት በ ህይወት ውስጥ የሚያጋጥሙ ችግሮችን ይፈታል ብለው ያስባሉ? | |  |  |  | |  | |  | |

አመሰግናለሁ!!
