# Supplementary material for: Willingness to work in rural areas and associated factors among graduating health students at the University of Gondar, northwest Ethiopia, 2021
Source: PLoS One. 2022 Oct 21;17(10):e0276594. doi: 10.1371/journal.pone.0276594 (PMC9586395; doi:10.1371/journal.pone.0276594)
Supplement: Attachment — Submitted filename: PONE-D-21-30311-reviewed.pdf [file pone.0276594.s003.pdf]

PLOS ONE

Willingness to work in rural areas and associated factors among graduating medical and health science students at the University of Gondar, northwest Ethiopia, 2021

--Manuscript Draft--

Manuscript Number:	PONE-D-21-30311
Article Type:	Research Article
Full Title:	Willingness to work in rural areas and associated factors among graduating medical and health science students at the University of Gondar, northwest Ethiopia, 2021
Short Title:	Willingness to work in rural areas and associated factors
Corresponding Author:	Azmeraw Ambachew Kebede, MSc<br>University of Gondar College of Medicine and Health Sciences<br>Gondar, Amhara regional State ETHIOPIA
Keywords:	Ethiopia, willingness, graduating students, medical and health sciences
Abstract:	Background<br>Many of the rural areas in developing countries are still need access to quality healthcare services. To ensure the fair distribution of a high-quality health workforce and the availability of health services, there is a need to assess the background barriers that explain why healthcare providers are not interested to work in rural areas, thereby setting strategies to achieve universal health coverage. Therefore, this study is aimed to assess willingness to work in rural areas and associated factors among medical and health science students at the University of Gondar.<br>Methods<br>An institution-based cross-sectional study was conducted at the University of Gondar from August 15 to 25. A total of 422 study participants were selected using a stratified simple random sampling technique. A pretested self-administered questionnaire was employed to collect the data. Data were entered into EPI DATA 4.6 and exported to SPSS 25 for further analysis. Multivariable logistic regression analysis was performed to identify factors associated with student's willingness to work in rural areas. The level of significance was decided based on the AOR with a 95 % confidence interval at a p-value of ≤ 0.05.<br>Results<br>In this study, it was found that medical and health science student's willingness to work in rural areas was 78.4% (95% CI: 74.3, 82.4). Being male (AOR = 2.15; 95% CI: 1.17, 3.94), having intention to continue with their profession (AOR = 2.5; 95% CI: 1.28, 4.86), having a favorable attitude towards working in rural areas (AOR = 7.32; 95% CI: 5.71, 18.65), and having a mother with no formal education (AOR = 2.23; 95% CI: 1.02, 4.85) and completing primary education (AOR = 2.69; 95% CI: 1.1, 6.61) were significantly associated with willingness to work in rural areas.<br>Conclusion<br>The willingness of students to work in rural areas was optimal. This calls for concerned bodies to create a conducive environment for male and female students to engage in rural areas without hesitation. It is also important to ensure that students are willing to work in the rural areas voluntarily, instead of working in rural areas due to a lack of employment.
Order of Authors:	Azmeraw Ambachew Kebede, MSc
	Blen Getachew, BSc
	Biruk Bizuneh, BSc
	Birhanu Ewunetu, BSc
	Dawit Kassahun, BSc
	Desalegn Fentahun, BSc
	Destaw Ademe, BSc
	Endeshaw Admasu Cherkos, Assistant professor

Powered by Editorial Manager® and ProduXion Manager® from Aries Systems Corporation

Additional Information:	
**Question**	**Response**
**Financial Disclosure**<br><br>Enter a financial disclosure statement that describes the sources of funding for the work included in this submission. Review the submission guidelines for detailed requirements. View published research articles from *PLOS ONE* for specific examples.<br><br>This statement is required for submission and **will appear in the published article** if the submission is accepted. Please make sure it is accurate.<br><br>**Unfunded studies**<br>Enter: *The author(s) received no specific funding for this work.*<br><br>**Funded studies**<br>Enter a statement with the following details:<br>• Initials of the authors who received each award<br>• Grant numbers awarded to each author<br>• The full name of each funder<br>• URL of each funder website<br>• Did the sponsors or funders play any role in the study design, data collection and analysis, decision to publish, or preparation of the manuscript?<br>• **NO** - Include this sentence at the end of your statement: *The funders had no role in study design, data collection and analysis, decision to publish, or preparation of the manuscript.*<br>• **YES** - Specify the role(s) played.<br><br>* typeset	The authors received no specific funds for this work
**Competing Interests**<br><br>Use the instructions below to enter a competing interest statement for this submission. On behalf of all authors, disclose any competing interests that could be perceived to bias this work—acknowledging all financial support and any other relevant financial or non-financial competing interests.	The authors declare that they have no conflict of interest in this work

This statement is required for submission and **will appear in the published article** if the submission is accepted. Please make sure it is accurate and that any funding sources listed in your Funding Information later in the submission form are also declared in your Financial Disclosure statement.<br><br>View published research articles from *PLOS ONE* for specific examples.<br><br>**NO authors have competing interests**<br><br>Enter: *The authors have declared that no competing interests exist.*<br><br>**Authors with competing interests**<br><br>Enter competing interest details beginning with this statement:<br><br>*I have read the journal's policy and the authors of this manuscript have the following competing interests: [insert competing interests here]*<br><br>* typeset	
**Ethics Statement**<br><br>Enter an ethics statement for this submission. This statement is required if the study involved:<br><br>• Human participants<br>• Human specimens or tissue<br>• Vertebrate animals or cephalopods<br>• Vertebrate embryos or tissues<br>• Field research<br><br>Write "N/A" if the submission does not require an ethics statement.<br><br>General guidance is provided below. Consult the submission guidelines for detailed instructions. **Make sure that all information entered here is included in the**	The ethical clearance letter was obtained from the School of Midwifery under the delegation of the Institutional Review Board (IRB) of the University of Gondar. A formal administrative approval letter was gained from the college of medicine and health sciences. After clearly explaining the purpose of the study for the students, anonymous written informed consent was obtained from each study participant. Interviewees have been given the right to refuse and withdraw from the study at any time

**Methods section of the manuscript.**<br><br>**Format for specific study types**<br><br>**Human Subject Research (involving human participants and/or tissue)**<br>• Give the name of the institutional review board or ethics committee that approved the study<br>• Include the approval number and/or a statement indicating approval of this research<br>• Indicate the form of consent obtained (written/oral) or the reason that consent was not obtained (e.g. the data were analyzed anonymously)<br><br>**Animal Research (involving vertebrate animals, embryos or tissues)**<br>• Provide the name of the Institutional Animal Care and Use Committee (IACUC) or other relevant ethics board that reviewed the study protocol, and indicate whether they approved this research or granted a formal waiver of ethical approval<br>• Include an approval number if one was obtained<br>• If the study involved *non-human primates*, add *additional details* about animal welfare and steps taken to ameliorate suffering<br>• If anesthesia, euthanasia, or any kind of animal sacrifice is part of the study, include briefly which substances and/or methods were applied<br><br>**Field Research**<br><br>Include the following details if this study involves the collection of plant, animal, or other materials from a natural setting:<br>• Field permit number<br>• Name of the institution or relevant body that granted permission	
**Data Availability**<br><br>Authors are required to make all data underlying the findings described fully available, without restriction, and from the time of publication. PLOS allows rare exceptions to address legal and ethical concerns. See the PLOS Data Policy and FAQ for detailed information.	Yes - all data are fully available without restriction

Powered by Editorial Manager® and ProduXion Manager® from Aries Systems Corporation

A Data Availability Statement describing where the data can be found is required at submission. Your answers to this question constitute the Data Availability Statement and **will be published in the article**, if accepted.<br><br>**Important:** Stating 'data available on request from the author' is not sufficient. If your data are only available upon request, select 'No' for the first question and explain your exceptional situation in the text box.<br><br>Do the authors confirm that all data underlying the findings described in their manuscript are fully available without restriction?	
**Describe where the data may be found in full sentences. If you are copying our sample text, replace any instances of XXX with the appropriate details.**<br><br>• If the data are **held or will be held in a public repository**, include URLs, accession numbers or DOIs. If this information will only be available after acceptance, indicate this by ticking the box below. For example: *All XXX files are available from the XXX database (accession number(s) XXX, XXX.*).<br>• If the data are all contained **within the manuscript and/or Supporting Information files**, enter the following: *All relevant data are within the manuscript and its Supporting Information files.*<br>• If neither of these applies but you are able to provide **details of access elsewhere**, with or without limitations, please do so. For example:<br><br>*Data cannot be shared publicly because of [XXX]. Data are available from the XXX Institutional Data Access / Ethics Committee (contact via XXX) for researchers who meet the criteria for access to confidential data.*<br><br>*The data underlying the results*	All relevant data are within the manuscript and its Supporting and Information files.

presented in the study are available from (include the name of the third party and contact information or URL).<br>• This text is appropriate if the data are owned by a third party and authors do not have permission to share the data.<br><br>* typeset	
Additional data availability information:	

Powered by Editorial Manager® and ProduXion Manager® from Aries Systems Corporation

Manuscript Click here to access/download;Manuscript;Manuscript.docx

RESEARCH ARTICLE

Willingness to work in rural areas and associated factors among graduating medical and health science students at the University of Gondar, northwest Ethiopia, 2021

Blen Getachew[1], Biruk Bizuneh[1], Birhanu Ewunetu[1], Dawit Kassahun[1], Dessalegn Fentahun[1], Destaw Ademe[1], Endeshaw Admasu Cherkos[2], Azmeraw Ambachew Kebede[3*]

[1]Department of General Midwifery, College of Medicine and Health Sciences, University of Gondar, Gondar Ethiopia; [2]Department of Women's and Family Health, School of Midwifery, College of Medicine and Health Sciences, University of Gondar, Gondar, Ethiopia; [3]Department of Clinical Midwifery, School of Midwifery, College of Medicine and Health Sciences, University of Gondar, Gondar, Ethiopia

[*] azmuzwagholic@gmail.com

Abstract

Background

Many of the rural areas in developing countries are still need access to quality healthcare services. To ensure the fair distribution of a high-quality health workforce and the availability of health services, there is a need to assess the background barriers that explain why healthcare providers are not interested to work in rural areas, thereby setting strategies to achieve universal health coverage. Therefore, this study is aimed to assess willingness to work in rural areas and associated factors among medical and health science students at the University of Gondar.

Methods

An institution-based cross-sectional study was conducted at the University of Gondar from August 15 to 25. A total of 422 study participants were selected using a stratified simple random sampling technique. A pretested self-administered questionnaire was employed to collect the data. Data were entered into EPI DATA 4.6 and exported to SPSS 25 for further analysis. Multivariable logistic regression analysis was performed to identify factors associated with student's willingness to work in rural areas. The level of significance was decided based on the AOR with a 95 % confidence interval at a p-value of ≤ 0.05.

Results

In this study, it was found that medical and health science student's willingness to work in rural areas was 78.4% (95% CI: 74.3, 82.4). Being male (AOR = 2.15; 95% CI: 1.17, 3.94), having intention to continue with their profession (AOR = 2.5; 95% CI: 1.28, 4.86), having a favorable attitude towards working in rural areas (AOR = 7.32; 95% CI: 5.71, 18.65), and having a mother with no formal education (AOR = 2.23; 95% CI: 1.02, 4.85) and completing primary education (AOR = 2.69; 95% CI: 1.1, 6.61) were significantly associated with willingness to work in rural areas.

Conclusion

The willingness of students to work in rural areas was optimal. This calls for concerned bodies to create a conducive environment for male and female students to engage in rural areas without

hesitation. It is also important to ensure that students are willing to work in the rural areas voluntarily, instead of working in rural areas due to a lack of employment.

Keywords: Ethiopia, willingness, graduating students, medical and health sciences

Introduction

The health workforce plays a key role in the provision of health. The equitable distribution of quality health personnel contributes significantly to ensure the availability of healthcare services, irrespective of their location. Besides, it helps in progressing towards the Universal Health Coverage (UHC) goal by facilitating access to quality healthcare services for all (1).

More than 4 million health workers are needed to fill the gap in the global health workforce, as estimated by the World Health Organization (WHO) (2). However, the geographical distribution of health workers is concentrated in affluent cities and regions worldwide, take no notice of rural areas. Regardless of the level of economic development and organization of the health system, this model exists in almost every country in the world, but this problem is particularly prominent in developing countries (3).

The shortage of health workers in rural areas has been identified as one of the biggest challenges for most countries to achieve the desired health-related goals. Developing countries suffer severely due to a significant shortage of health works proportional to the population (4). Ethiopia is also one of the victims of this challenge, despite having about 84% rural population (5). One of the challenges to provide at least basic health services is the lack of trained health workers (6). In connection to this, the WHO recommends that at least 4.45 doctors, nurses, and midwives per 1000 population are needed to achieve the minimum levels of key health interventions. However, Africa has a 2.2 health workforce per 1000 population, which is often challenging to achieve the global goals (1).

In Ethiopia, the overall health professionals to population ratio were 1 per 1,000 population in 2018 (7), which is far below the minimum number estimated by the WHO. Hence, the healthcare system still suffers from the shortage and the uneven distribution of healthcare workers between the urban and rural populations (8). The shortage of health staff also has a significant impact on the healthcare delivery system such as the provision of essential life-saving interventions such as providing Expanded Program on Immunization (EPI), clean water, safe pregnancy, and

childbirth services, and access to Human Immunodeficiency deficiency Virus/Acquired Immunodeficiency Syndrome (HIV/AIDS), tuberculosis (TB), and malaria treatment (9).

The Sustainable Development Goals (SDG) by United Nations (UN) member states in 2015, particularly goal 3 aim to ensure health and wellbeing for all. Target 3.1 specifically aims to reduce the Maternal Mortality Ratio (MMR) to less than 70 per 100,000, thereby saving the lives of one million mothers (10). In addition, 405,000 people died of malaria in 2018, 94% of which occurred in Africa (11). Moreover, an estimated 1.3 million TB-related deaths were recorded in 2017; additional 300,000 deaths among people living with HIV/AIDS (12). All these burdens are more common in rural areas. Hence, assigning adequate healthcare providers to these highly vulnerable groups will greatly help reduce the deaths of many lives.

Most of the morbidity and mortality are scattered in the rural areas. Thus, the willingness of healthcare providers to work in rural areas is crucial to basically alleviate the problem. However, most health care providers are reluctant to work in rural settings. Some of the commonly cited reasons for not working in these areas are perceived low salary, unavailability of professional standards, unsatisfactory working environment, lack of access to skilled colleagues, and modern equipment (9). Besides, some others stated that learning and training opportunities are scarce while they get employed in the rural areas (13). Furthermore, lack of the expected living conditions including electricity, water supply, and schools for their children, and having a plan to migrate out of their country after graduation are some of the reasons that lower the willingness to work in rural areas (13,14). In this regard, it is necessary to set problem-solving strategies by providing adequate remuneration for their work, better professional training, and educational opportunities, and identifying effective and efficient human and non-human resources to meet the individual health needs of the underserved rural population.

Medical and health science student's desire to go to the countryside will play an important role for the rural community that has been denied access to health services. Previous empirical evidence on student's willingness to work in rural areas revealed that China-52% (15), India-45% (16), Nigeria-33.5% (17), Ghana-55.4% (18), and Ethiopia-29.7% (19). Being a male student (15,18), having a family member of lower academic attainments and lower socioeconomic status (20), rural background (21), and having a good attitude towards the rural community (22) had a positive impact on students willing to work in the rural areas.

Although the health workforce in Ethiopia is growing, it is still less than half of the minimum number estimated by WHO. Increasing the willingness of graduating students by designing effective strategies will play a key role in reducing the burden of shortage of health care workers in the rural areas, thereby achieving the optimal health of the marginalized rural population. Therefore, this study was aimed to assess willingness to work in the rural areas and associated factors among graduating medicine and health science students at the University of Gondar, northwest Ethiopia.

Methods and materials

Study design, period, and study area

An institution-based cross-sectional study was conducted from August 1 to 25, 2021. The study was conducted at the University of Gondar, College of Medicine and Health Sciences, which is found at Gondar city, northwestern Ethiopia. The college was established in 1954 and is considered one of the oldest and most famous universities in Ethiopia. There are 12 fields of study in health and medicine such as Nursing, Midwifery, Clinical laboratory, Pharmacy, Anesthesia, Psychiatry, Health informatics, Physiotherapy, Optometry, Environmental and occupational health, Public health officer (HO), and Medicine. Currently, there are 1068 graduating students from the 12 departments on September 2021.

Study population

All graduating medical and health science students during the study period were the study population.

Sample size determination and sampling procedure

The sample size for this study was calculated based on the assumptions of the single population proportion formula by considering the following issues: proportion of student's willingness to work in the rural area-50% ($p = 0.5$), level of significance- 5 % ($\alpha = 0.05$), Z $\alpha/2$ - 1.96, and margin of error - 5% ($\alpha = 0.05$), and non - response rate ~~10.~~ Therefore, sample size was calculated as follows

$$N = \frac{(Z\alpha/2)^2 * p(1-p)}{d^2} = \quad N = \frac{(1.96)^{2} * 0.5(1-0.5)}{(0.05)2} = 384.$$

The minimum adequate sample size was found to be 422, after adding a 10% non-response rate. There are 12 departments at the University of Gondar, college of medicine, and health sciences. All departments were included in the study. The lists of students were obtained from the registrar's office and the sampling frame was prepared by ordering the lists of students. Then, the total sample size was distributed proportionally to each department. Finally, the participants were selected using a simple random sampling technique using a table of random generation **(Figure 1).**

Variables of the study

The willingness of students to work in the rural areas was the outcome variable, whereas age, sex, place of origin, religion, professional category, paternal and maternal educational status, and paternal and maternal occupation, and personal behaviors like exposure to addictive substances, attitude to work in the rural area, and plan to emigrate.

Measurements

Willing to work in rural areas: Students were asked whether they have a willingness to work in rural areas or not after graduation. Students who said "yes" when they are asked, whether they are willing to work in the rural areas after graduation or not, was considered has a willingness to work in the rural areas (18).

Favorable attitude towards working in rural areas: Based on the summative score of variables designed to assess attitude-related questions, those students who answered above the mean are considered as having a favorable attitude (16).

Data collection tools and procedures

The data collection tool was developed by reviewing the literature (15,16,18,19) and data were collected using a structured, pretested, and self-administered questionnaire. The questionnaire contains socio-demographic characteristics and academic-related characteristics. Six BSc in midwifery graduating students and 1 MSc in Midwifery holder, trained about the interview technique, collect and supervise the data collection process, respectively.

Data quality control

To ensure consistency and understandability, the questionnaire was initially prepared in English and translated to the local Amharic language and back to English. A pretest was done on 5% of the calculated sample size at Bahir Dar University to check the response, language intelligibility, and relevance of the questionnaire. The data collectors and supervisors were trained for a day regarding the overall data collection process. At the time of actual data collection, the collected data was checked daily by the supervisor for completeness.

Data processing and analysis

Data were entered into EPI DATA 4.6 and exported to SPSS version 25 for further cleaning and analysis. Tables, figures, mean, and proportions are used to state the descriptive statistics of the study participants. Binary logistic regression was fitted to identify independent predictors, and variables with a p-value of less than 0.2 were included in the multivariable logistic regression to control potential confounding factors. The chi-square assumption was tested and Hosmer-Lemeshow was used to test the model's goodness of fit. In addition, variance inflation factor (VIF), and standard error were used to screen multi-collinearity among independent variables. In the multivariable logistic regression analysis, a p-value of ≤ 0.05 and 95% CI for the adjusted odds ratio (AOR) were used to determine the significant association between willingness to work in rural areas and the explanatory variables.

Ethical considerations

The ethical clearance letter was obtained from the School of Midwifery under the delegation of the Institutional Review Board (IRB) of the University of Gondar. A formal administrative approval letter was gained from the college of medicine and health sciences. After clearly explaining the purpose of the study for the students, anonymous written informed consent was obtained from each study participant. Interviewees have been given the right to refuse and withdraw from the study at any time.

Result

Socio-demographic characteristics of study participants

A total of 422 study participants were recruited but only 402 of them completed the study, giving a response rate of 95.26%. The mean age of the students was 23.9 years old (SD ± 2.28) and the majority of students were in the age group of < 25 years. Nearly two-thirds (62.4%) of the study participants were males. About 29.4% of the participants were medical students and 46% of them were from urban areas **(Table 1).**

Academic, medical, and behavior related characteristics

From the total study participants, nearly three-fourths (73.9%) believed that more job opportunities are available in the urban areas. About 58.5% of the study participants joined their first departmental choice and have got information regarding the department before campus entry. More than two-thirds (68.4%) and fourth-fifths (81.1%) of the study subjects had a favorable attitude towards working in rural areas and intended to stay in their profession, respectively **(Table 2).**

Willingness to work in rural areas and associated factors among graduating students

The willingness of students to work in rural areas was 78.4% (95% CI: 74.3, 82.4). Besides, the multivariable logistic regression analysis revealed that being male, had an intention to continue with the profession, had a mother with no formal education and attending primary education, and having a favorable attitude towards working in rural areas were variables having a significant association with willingness to work in rural areas.

Hence, the odds of having a good willingness to work in rural areas was two times higher among male students (AOR = 2.15; 95% CI: 1.17, 3.94) as compared to female students. Similarly, students who had an intention to continue with their profession were 2.5 times higher (AOR = 2.5; 95% CI: 1.28, 4.86) compared with those students who hadn’t have intention. Likewise, the likelihood of having a willingness to work in rural areas was 7.32 times higher (AOR = 7.32; 95% CI: 5.71, 18.65) among students who had a favorable attitude towards working in rural areas as compared to their counterparts. Lastly, students who had a mother with no formal education and attending primary education were 2.23 (AOR = 2.23; 95% CI: 1.02, 4.85) and 2.69 times (AOR = 2.69; 95% CI: 1.1, 6.61) more likely to have had a willingness to work in

rural areas as compared to students who had a mother ~~attending~~ college and above education, respectively (**Table 3**).

Perceived reasons for not willing to work in rural areas

About 21.6% (87) of the study participants were not willing to work in rural areas after graduation. The frequently mentioned reasons by the students for not have a willingness to work in rural areas include lack of infrastructure in rural areas, not to be far from family members, and peace and security issues **(Figure 2).**

Discussion

This study aimed to assess the willingness to work in rural areas, and associated factors among graduating medical and health science students at the University of Gondar, northwest Ethiopia. Thus, more than three-fourths of students were willing to work in rural areas. In addition, being male, having an intention to continue with their profession, having a favorable attitude towards working in rural areas, and having a mother with no formal education and attending primary education were factors associated with student's willingness to work in rural areas.

This study found that student's willingness to work in rural areas was 78.4%, which was far higher than studies conducted in Egypt-52.3% (13), China-52% (15), India-45% (16), Nigeria-33.5% (17), Ghana-55.4% (18), and Ethiopia-29.7% (19). The possible explanation for the variation might be differences in socio-demographic contexts and time gaps. More than 80% of Ethiopia's population lives in rural areas, which may need more health workers, and new job opportunities in the health care system are available there. Besides, there may not be many health professionals before, and no one wants to go to the countryside to work because there was no shortage of jobs in urban areas previously. Nowadays, however, there may be a higher number of health professionals than ever before, and jobs may not be easy to find, especially in Ethiopia, so newly graduating students may want to work in rural areas unreservedly. In light of this, the Ethiopian government and other stakeholders should address and implement conditions for job creations for medical and health science students, and arrange opportunities that will favor students to work in rural areas in all aspects. In turn, this idea will reduce the student's intention to leave their profession and burnout in the workplace.

Regarding factors associated with willingness to work in rural areas, this study affirmed that the sex of students affects their willingness to work in rural areas. Accordingly, male students were two times more likely to have had a good willingness to work in rural areas as compared to their female counterparts. This finding is consistent with studies conducted in Egypt and Ghana (15,18). This can be explained by the fact that male students might cope with problems that are considered to be present in rural areas, such as transportation problems, lack of electricity, and water, etc. This is because when there is a lack of transportation and a scarcity of daily consumption, there may be a need to walk to the city on foot, and females may be afraid that they will be in trouble. However, this finding contradicts a study conducted in China that founds female students are more intend to work in rural areas (20). This could be justified by differences in the country's infrastructure and culture of the community. In Ethiopia, the infrastructure might not be propitious for females like that of China. Besides, female students might not be willing to be far from families because of the perception of the community, in which believing females might not being able to deal with the difficult environment they might face.

This study revealed that students who had a mother with no formal education and attending primary education were 2.23 and 2.69 times more likely to have had a willingness to work in rural areas as compared to students who had a mother attending college and above education. This finding is in agreement with a study conducted in China (20). This could be explained by parents might have a big influence on shaping and directing the attitude and behaviors of their children, including educations what to learn and work something else. Besides, those students who had a family with less educational achievement are expected to be less affluent. Thus, to support the household economy, students will have a willingness to work in rural areas irrespective of preconditions.

In this study, the odds of willingness to work in rural areas was two times higher among students who had plans to continue with their profession as compared to those students who hadn't intended to continue with the profession that they have currently. A study conducted in Nigeria reported a similar result (23). This could be explained by the fact that students who intend to continue with their profession may initially join the department by their choice and could have a strong commitment to work in any part of the country. On the other hand, students may not have the interest to continue with their current profession due to different reasons like a plan to

immigrate to foreign counties and engage in other business. Because those students with no intention to continue with their profession might have a plan to participate in other income-generating activities and plan to change their profession due to lack of interest in the department after they engaged in. In this regard, the Ethiopian government should pay attention to the healthcare providers by settling different encouraging undertakings such as crafting job opportunities, evenhanded salary for their work, and timely education chances for their vertical growth.

This study also was found that students' attitudes towards rural work affected their willingness to work in rural areas after graduation. Accordingly, students with a positive attitude towards work in rural areas were seven times more likely than students with unfavorable attitudes. This result is consistent with a study conducted in Ethiopia (22). The attitude of an individual had a direct impact in determining responses to situations, psychological preparations, and influencing responses to all situations around them (16). Thus, students who have a positive attitude to the need for rural community support and access to equitable health care may want to work in rural areas unconditionally. They might also be happy to help people, and they may be able to fulfill their good wishes by serving the rural people, where highly in need of healthcare access. Lastly, we are pleased to acknowledge some of the limitations of this study. Due to the cross-sectional nature of the study design, it may not be possible to know the exact effect of the identified factors on the outcome variable. Moreover, since the questionnaire was self-administered, response bias might be introduced. However, before data collection, students were told that their participation in the research is significant and that they would fill in the required information honestly for the success of the research.

Conclusion

More than three-quarters of medical and health science students were willing to work in rural areas. Being a male student, having the intention to stay in their profession, having a good attitude towards working in rural areas, having a good attitude towards working in rural areas, and students having a mother of low academic achievement was observed to affect the student's willingness to work in the rural areas. Thus, this calls for health policymakers and other stakeholders to give attention to arrange all-encompassing job opportunities in the rural areas

that will favor both males and females. In addition, it is important to build a good view and understanding for students about rural areas during their campus life. Moreover, benefits improvement is essential for students to stay in their profession without getting disappointed and planned to leave their profession.

Supporting information

S1 Questionnaire: English and Amharic version.

S2 Dataset: SPSS data.

Authors' Contributions

Conceptualization: Azmeraw Ambachew Kebede

Data curation: Blen Getachew, Biruk Bizuneh, Birhanu Ewunetu, Dawit Kassahun, Dessalegn Fentahun, Destaw Ademe, Endeshaw Admasu Cherkos, Azmeraw Ambachew Kebede

Formal analysis: Blen Getachew, Biruk Bizuneh, Birhanu Ewunetu, Dawit Kassahun, Dessalegn Fentahun, Destaw Ademe, Endeshaw Admasu Cherkos, Azmeraw Ambachew Kebede

Investigation: Blen Getachew, Biruk Bizuneh, Birhanu Ewunetu, Dawit Kassahun, Dessalegn Fentahun, Destaw Ademe, Endeshaw Admasu Cherkos, Azmeraw Ambachew Kebede

Methodology: Blen Getachew, Biruk Bizuneh, Birhanu Ewunetu, Dawit Kassahun, Dessalegn Fentahun, Destaw Ademe, Endeshaw Admasu Cherkos, Azmeraw Ambachew Kebede
Validation: Blen Getachew, Biruk Bizuneh, Birhanu Ewunetu, Dawit Kassahun, Dessalegn Fentahun, Destaw Ademe, Endeshaw Admasu Cherkos, Azmeraw Ambachew Kebede

Visualization: Blen Getachew, Biruk Bizuneh, Birhanu Ewunetu, Dawit Kassahun, Dessalegn Fentahun, Destaw Ademe, Endeshaw Admasu Cherkos, Azmeraw Ambachew Kebede

Writing-original draft: Blen Getachew and Azmeraw Ambachew Kebede

Writing- review and editing: Blen Getachew, Biruk Bizuneh, Birhanu Ewunetu, Dawit Kassahun, Dessalegn Fentahun, Destaw Ademe, Endeshaw Admasu Cherkos, Azmeraw Ambachew Kebede

Acknowledgments

We would like to thank the University of Gondar for providing study ethical clearance to conduct this study. Our gratitude also goes to all data collectors and study participants. We are glad to Hospitals in Gondar province for writing permission letter.

Data Availability Statement

All relevant data are within the manuscript and its Supporting and Information files.

Funding: The authors received no specific funds for this work.

Competing interests: The authors declare that they have no conflict of interest in this work

Abbreviations

AOR; Adjusted Odds Ratio, CI; Confidence Interval, COR Crude Odds Ratio, SPSS; Statistical Package for Social Science, SSA; Sub-Saharan Africa, WHO; World Health Organization

Table 1: Socio-demographic characteristics of study participants in the University of Gondar, college of medicine and health sciences, northwest Ethiopia, 2021 (n = 402)

Charactestics	Frequency	Percentage (%)
**Age in year**		
>25	283	70.4
≥25	119	29.4
**Sex**	251	62.4
Male	151	37.6
Female		
**Place of birth**		
Urban	185	46
Semi-urban	59	14.7
Rural	158	39.3
**Religion**		

Orthodox Christian	344	85.6
Protestant	42	10.4
Others[a]	16	4
**Current marital status**		
Married	19	4.7
In relationship	31	7.7
Single	352	87.6
**Average monthly pocket money**		
< 500	70	17.4
500-999	108	26.7
≥ 1000	224	55.7
**Department**		
Medicine	118	29.4
Nursing	43	10.7
Midwifery	33	8.2
Public health officer	32	8
Psychiatry	17	4.2
Environmental and occupational health	21	5.2
Optometry	17	4.2
Physiotherapy	17	4.2
Health informatics	20	5
Anesthesia	18	4.5
Pharmacy	35	8.7
Medical laboratory	31	7.7

**Average monthly income of the family**		
<1500	14	3.5
1501-5000	115	28.6
≥ 5001	273	67.9
**Mothers educational status**		
No formal education	159	39.6
Primary education	83	20.6
Secondary education	67	16.7
Diploma and above	93	23.1
**Mother's occupation**		
Housewife and/or farmer	229	57
Government employee	67	16.7
Self employed	47	11.7
Merchant	57	14.2
Others[b]	2	0.5
**Father's educational status**		
No formal education	110	27.4
Primary	120	29.9
Secondary	47	11.7
Diploma and above	125	31.1
**Fathers occupation**		
Farmer	168	41.8
Merchant	74	18.4
Government employed	105	26.1

Self employed	53	13.2
Others[b]	2	0.5

a = Muslim and catholic, b = retired

Table 2: Academic, medical, and behavioral related characteristics of study participants in the University of Gondar, college of medicine and health sciences, northwest Ethiopia, 2021 (n = 402)

Charactestics	Frequency	Percentage (%)
**History of chat chewing**		
Yes	33	91.8
No	369	8.2
**History of cigarette smoking**		
Yes	23	5.7
No	379	94.3
**Ever drunk alcohol**		
Yes	178	55.7
No	224	44.3
**Getting information about the department before campus entry**		
Yes	240	59.7
No	162	40.3
**Source of information (n = 240)**		
Friends	116	48.3
Healthcare providers	17	7.1
Families	61	25.4

Mass media	46	19.2
**Is the department your first choice**		
Yes	235	58.5
No	167	41.5
**Intention to continue with the profession**		
Yes	329	81.1
No	73	18.2
**Attitude towards working in rural areas**		
Favorable	275	68.4
Unfavorable	126	31.1
**Where did you think jobs are available**		
Urban	297	73.9
Rural	105	26.1
**Do you have any known medical disorder**		
Yes	384	95.5
No	18	4.5

Table 3: Bivariable and multivariable logistic regression analysis of factors associated with graduating students willingness to work in rural areas in the University of Gondar, northwest Ethiopia, 2021 (n = 402)

Variables	Willingness to work in rural areas		COR (95% CI)	AOR (95% CI)
	Yes	No		
**Sex**				
Male	214	37	2.86 (1.76, 4.65)	**2.15 (1.17, 3.94)***

Female	101	50	1	1
**Age in year**				
< 25	213	70	1	1
≥ 25	102	17	1.97 (1.10, 3.52)	1.35 (0.66, 2.75)
**Place of birth**				
Urban	132	53	1	1
Semi-urban	46	13	1.42 (0.71, 2.84)	0.92 (0.36, 2.31)
Rural	137	21	2.62 (1.49, 4.58)	1.61 (0.63, 4.12)
**Intention to continue with the profession**				
Yes	267	62	2.24 (1.28, 3.91)	**2.50 (1.28, 4.86)***
No	48	25	1	1
**Mother's educational status**				
No formal education	137	22	2.54 (1.35,4.81)	**2.23 (1.02, 4.85)***
Primary education	72	11	2.68 (1.23, 5.82)	**2.69 (1.1, 6.61)***
Secondary education	40	27	0.6 (0.31, 1.17)	0.58 (0.268, 1.27)
Diploma and above	66	27	1	1
**Father's educational status**				
No formal education	96	14	3.35 (1.70, 6.56)	3.35 (1.00, 10.36)
Primary education	102	18	2.76 (1.48, 5.16)	1.62 (0.66, 4.01)
Secondary education	33	14	1.15 (0.55, 2.38)	1.03 (0.39, 2.72)
Diploma and above	84	41	1	1
**Attitude towards working in the rural area**				

Favorable	249	26	8.7 (5.10, 14.85)	**7.32 (5.71,18.65)****
Unfavorable	66	60		1

Notes: * $P \leq 0.05$, ** $P \leq 0.001$

Abbreviations: AOR, adjusted odds ratio; COR, crude odds ratio; CI, confidence interval; 1, reference category

Reference

1. Health workforce requirements for universal health coverage and the sustainable development goals ((Human Resources for Health Observer, 17). World health Organization. 2016;

2. Working Together for Health- World Health report. World Health Organization. 2011;

3. Asante AD, Zwi AB, AD A, AB Z. Factors influencing resource allocation decisions and equity in the health system of Ghana. 2009 May;123(5):371–7.

4. Liu JX, Goryakin Y, Maeda A, Bruckner T, Scheffler R. Global Health Workforce Labor Market Projections for 2030. Hum Resour Health. 2017;15(1).

5. Assefa Y, Hill PS, Gilks CF, Admassu M, Tesfaye D, Van Damme W. Primary health care contributions to universal health coverage, Ethiopia. Bull World Health Organ. 2020;98(12):894-905A.

6. Mengistu BS, Vins H, Kelly CM, McGee DR, Spicer JO, Derbew M, et al. Student and faculty perceptions on the rapid scale-up of medical students in Ethiopia. BMC Med Educ [Internet]. 2017;17(1):1–8. Available from: http://dx.doi.org/10.1186/s12909-016-0849-0

7. Health Sector Transformation Plan II 2020/2021-2024/2025. Ethiop Minist Heal. 2021;25(February):1–128.

8. Bradley E, Thompson JW, Byam P, Webster TR, Zerihun A, Alpern R, et al. Access and quality of rural healthcare: Ethiopian Millennium Rural Initiative. Int J Qual Heal Care. 2011;23(3):222–30.

9. Girasek E, Eke E, Szócska M. Analysis of a survey on young doctors' willingness to work in rural Hungary. Hum Resour Heal 2010 81. 2010 May;8(1):1–6.

10. Final list of proposed Sustainable Development Goal indicators. Report of the Inter-Agency and Expert Group on Sustainable Development Goal Indicators (E/CN.3/2016/2/Rev.1). 2016; Available from: https://sustainabledevelopment.un.org/content/documents/11803Official-List-of-Proposed-SDG-Indicators.pdf

11. Regional and global trends in burden of malaria cases and deaths. World Health

Organization malaria report 2019. Available from: https://www.who.int/news-room/feature-stories/detail/world-malaria-report-2019.

12. Global tuberculosis report 2018. Geneva: World Health Organization; 2018. Licence: CC BY-NC-SA 3.0 IGO.

13. Mohamed AM. Willingness and professional motivations of medical students to work in rural areas: a study in Alexandria, Egypt. Healthc Low-resource Settings. 2013 Mar;1(1):e4–e4.

14. Deressa W, Azazh A. Attitudes of undergraduate medical students of Addis Ababa University towards medical practice and migration, Ethiopia. BMC Med Educ. 2012;12(1).

15. Liu J, Zhu B, Mao Y. Association between rural clinical clerkship and medical students' intentions to choose rural medical work after graduation: A cross-sectional study in western China. PLoS One. 2018 Apr;13(4):e0195266.

16. Rajesh Kumar Singh, CMS Rawat SP, Department. Attitude of medical students toward serving in rural areas and its determinants: a cross-sectional study from Uttarakhand. Int J Med Sci Public Heal. 2015;4(6):814.

17. Ndu AC, Aniwada EC, Umeh EN, Uzo NM, Uzoma CE. Perception and willingness of medical students towards working in rural communities. Niger J Med. 2019 Nov;28(3):275–80.

18. Agyei-Baffour P, Kotha SR, Johnson JC, Gyakobo M, Asabir K, Kwansah J, et al. Willingness to work in rural areas and the role of intrinsic versus extrinsic professional motivations - A survey of medical students in Ghana. BMC Med Educ. 2011;11(1):0–7.

19. Assefa T, Haile Mariam D, Mekonnen W, Derbew M. Medical students' career choices, preference for placement, and attitudes towards the role of medical instruction in Ethiopia. BMC Med Educ. 2017;17(1):1–10.

20. Liu J, Zhang K, Mao Y. Attitude towards working in rural areas: a cross-sectional survey of rural-oriented tuition-waived medical students in Shaanxi, China. BMC Med Educ 2018 181. 2018 May;18(1):1–11.

21. Serneels P, Montalvo JG, Pettersson G, Lievens T, Buterae JD, Kidanu A. Who wants to work in a rural health post? The role of intrinsic motivation, rural background and faith-based institutions in Ethiopia and Rwanda. Bull World Health Organ. 2010;88(5):342–9.

22. Deressa W, Azazh A, file:///C:/Users/Bell/Downloads/9789240005105-eng.pdfDeressa W, Azazh A. Attitudes of undergraduate medical students of Addis Ababa University towards medical practice and migration, Ethiopia. BMC Med Educ. 2012;12(1).

23. Article O. Emigration Plans after Graduation of Clinical Medical Students of Ebonyi State University Abakaliki, Nigeria: Implications for Policy. 2020;995–1003.

 Click here to access/download;Figure;List of Figures.tif

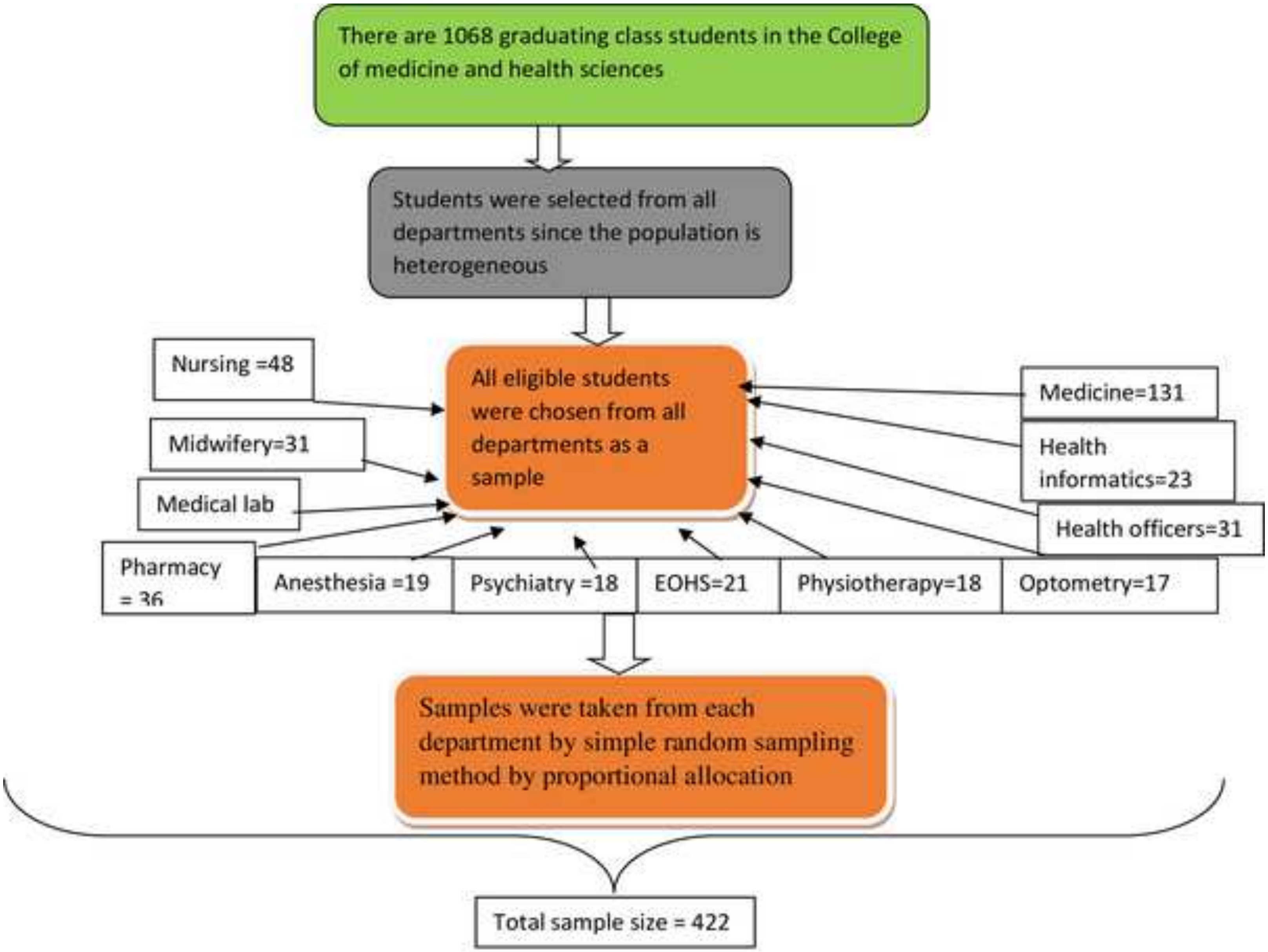

Figure 1: Schematic presentation of the sampling procedure for the study done on willingness to work in rural areas and associated factors among graduating medicine and health science students at university of Gondar, 2021

Questionnaire

Click here to access/download
Supporting Information
English and amhraric version questionnaire.docx

SPSS data

Click here to access/download
Supporting Information
SPSS data.sav
