# Supplementary material for: Willingness to work in rural areas and associated factors among graduating health students at the University of Gondar, northwest Ethiopia, 2021
Source: PLoS One. 2022 Oct 21;17(10):e0276594. doi: 10.1371/journal.pone.0276594 (PMC9586395; doi:10.1371/journal.pone.0276594)
Supplement: Attachment — Submitted filename: PONE-D-21-30311_R1_reviewercomments.pdf [file pone.0276594.s005.pdf]

PLOS ONE

# Willingness to work in rural areas and associated factors among graduating medical and health science students at the University of Gondar, northwest Ethiopia, 2021

--Manuscript Draft--

| | |
|---|---|
| Manuscript Number: | PONE-D-21-30311R1 |
| Article Type: | Research Article |
| Full Title: | Willingness to work in rural areas and associated factors among graduating medical and health science students at the University of Gondar, northwest Ethiopia, 2021 |
| Short Title: | Willingness to work in rural areas and associated factors |
| Corresponding Author: | Azmeraw Ambachew Kebede, MSc<br>University of Gondar College of Medicine and Health Sciences<br>Gondar, Amhara regional State ETHIOPIA |
| Keywords: | Ethiopia, willingness, graduating students, medical and health sciences |
| Abstract: | Background<br>Many of the rural areas in developing countries are still need access to quality healthcare services. To ensure the fair distribution of a high-quality health workforce and the availability of health services, there is a need to assess the background barriers that explain why healthcare providers are not interested to work in rural areas, thereby setting strategies to achieve universal health coverage. Therefore, this study is aimed to assess willingness to work in rural areas and associated factors among medical and health science students at the University of Gondar.<br>Methods<br>An institution-based cross-sectional study was conducted at the University of Gondar from August 15 to 25. A total of 422 study participants were selected using a stratified simple random sampling technique. A pretested self-administered questionnaire was employed to collect the data. Data were entered into EPI DATA 4.6 and exported to SPSS 25 for further analysis. Multivariable logistic regression analysis was performed to identify factors associated with student's willingness to work in rural areas. The level of significance was decided based on the AOR with a 95 % confidence interval at a p-value of ≤ 0.05.<br>Results<br>In this study, it was found that medical and health science student's willingness to work in rural areas was 78.4% (95% CI: 74.3, 82.4). Being male (AOR = 2.15; 95% CI: 1.17, 3.94), having intention to continue with their profession (AOR = 2.5; 95% CI: 1.28, 4.86), having a favorable attitude towards working in rural areas (AOR = 7.32; 95% CI: 5.71, 18.65), and having a mother with no formal education (AOR = 2.23; 95% CI: 1.02, 4.85) and completing primary education (AOR = 2.69; 95% CI: 1.1, 6.61) were significantly associated with willingness to work in rural areas.<br>Conclusion<br>The willingness of students to work in rural areas was optimal. This calls for concerned bodies to create a conducive environment for male and female students to engage in rural areas without hesitation. It is also important to ensure that students are willing to work in the rural areas voluntarily, instead of working in rural areas due to a lack of employment. |
| Order of Authors: | Azmeraw Ambachew Kebede, MSc |
| | Blen Getachew, BSc |
| | Biruk Bizuneh, BSc |
| | Birhanu Ewunetu, BSc |
| | Dawit Kassahun, BSc |
| | Desalegn Fentahun, BSc |
| | Destaw Ademe, BSc |
| | Endeshaw Admasu Cherkos, Assistant professor |

*Powered by Editorial Manager® and ProduXion Manager® from Aries Systems Corporation*

| Response to Reviewers: | Uploaded separately |
|---|---|
| Additional Information: | |
| Question | Response |
| **Financial Disclosure**<br><br>Enter a financial disclosure statement that describes the sources of funding for the work included in this submission. Review the submission guidelines for detailed requirements. View published research articles from *PLOS ONE* for specific examples.<br><br>This statement is required for submission and **will appear in the published article** if the submission is accepted. Please make sure it is accurate.<br><br>**Unfunded studies**<br>Enter: *The author(s) received no specific funding for this work.*<br><br>**Funded studies**<br>Enter a statement with the following details:<br>• Initials of the authors who received each award<br>• Grant numbers awarded to each author<br>• The full name of each funder<br>• URL of each funder website<br>• Did the sponsors or funders play any role in the study design, data collection and analysis, decision to publish, or preparation of the manuscript?<br>• **NO** - Include this sentence at the end of your statement: *The funders had no role in study design, data collection and analysis, decision to publish, or preparation of the manuscript.*<br>• **YES** - Specify the role(s) played.<br><br>* typeset | The authors received no specific funds for this work |
| **Competing Interests**<br><br>Use the instructions below to enter a competing interest statement for this submission. On behalf of all authors, disclose any competing interests that could be perceived to bias this work—acknowledging all financial support and any other relevant financial or non-financial competing interests. | The authors declare that they have no conflict of interest in this work |

Powered by Editorial Manager® and ProduXion Manager® from Aries Systems Corporation

| | |
|---|---|
| This statement is required for submission and **will appear in the published article** if the submission is accepted. Please make sure it is accurate and that any funding sources listed in your Funding Information later in the submission form are also declared in your Financial Disclosure statement.<br><br>View published research articles from *PLOS ONE* for specific examples.<br><br>**NO authors have competing interests**<br><br>Enter: *The authors have declared that no competing interests exist.*<br><br>**Authors with competing interests**<br><br>Enter competing interest details beginning with this statement:<br><br>*I have read the journal's policy and the authors of this manuscript have the following competing interests: [insert competing interests here]*<br><br>* typeset | |
| **Ethics Statement**<br><br>Enter an ethics statement for this submission. This statement is required if the study involved:<br><br>• Human participants<br>• Human specimens or tissue<br>• Vertebrate animals or cephalopods<br>• Vertebrate embryos or tissues<br>• Field research<br><br>Write "N/A" if the submission does not require an ethics statement.<br><br>General guidance is provided below. Consult the submission guidelines for detailed instructions. **Make sure that all** | The ethical clearance letter was obtained from the School of Midwifery under the delegation of the Institutional Review Board (IRB) of the University of Gondar. A formal administrative approval letter was gained from the college of medicine and health sciences. After clearly explaining the purpose of the study for the students, anonymous written informed consent was obtained from each study participant. Interviewees have been given the right to refuse and withdraw from the study at any time |

Powered by Editorial Manager® and ProduXion Manager® from Aries Systems Corporation

| | |
|---|---|
| **information entered here is included in the Methods section of the manuscript.**<br><br>**Format for specific study types**<br><br>**Human Subject Research (involving human participants and/or tissue)**<br>• Give the name of the institutional review board or ethics committee that approved the study<br>• Include the approval number and/or a statement indicating approval of this research<br>• Indicate the form of consent obtained (written/oral) or the reason that consent was not obtained (e.g. the data were analyzed anonymously)<br><br>**Animal Research (involving vertebrate animals, embryos or tissues)**<br>• Provide the name of the Institutional Animal Care and Use Committee (IACUC) or other relevant ethics board that reviewed the study protocol, and indicate whether they approved this research or granted a formal waiver of ethical approval<br>• Include an approval number if one was obtained<br>• If the study involved *non-human primates*, add *additional details* about animal welfare and steps taken to ameliorate suffering<br>• If anesthesia, euthanasia, or any kind of animal sacrifice is part of the study, include briefly which substances and/or methods were applied<br><br>**Field Research**<br><br>Include the following details if this study involves the collection of plant, animal, or other materials from a natural setting:<br>• Field permit number<br>• Name of the institution or relevant body that granted permission | |
| **Data Availability**<br><br>Authors are required to make all data underlying the findings described fully available, without restriction, and from the time of publication. PLOS allows rare exceptions to address legal and ethical | Yes - all data are fully available without restriction |

| | |
|---|---|
| concerns. See the PLOS Data Policy and FAQ for detailed information.<br><br>A Data Availability Statement describing where the data can be found is required at submission. Your answers to this question constitute the Data Availability Statement and **will be published in the article**, if accepted.<br><br>**Important:** Stating 'data available on request from the author' is not sufficient. If your data are only available upon request, select 'No' for the first question and explain your exceptional situation in the text box.<br><br>Do the authors confirm that all data underlying the findings described in their manuscript are fully available without restriction? | |
| **Describe where the data may be found in full sentences. If you are copying our sample text, replace any instances of XXX with the appropriate details.**<br><br>• If the data are **held or will be held in a public repository**, include URLs, accession numbers or DOIs. If this information will only be available after acceptance, indicate this by ticking the box below. For example: *All XXX files are available from the XXX database (accession number(s) XXX, XXX.*).<br>• If the data are all contained **within the manuscript and/or Supporting Information files**, enter the following: *All relevant data are within the manuscript and its Supporting Information files.*<br>• If neither of these applies but you are able to provide **details of access elsewhere**, with or without limitations, please do so. For example:<br><br>*Data cannot be shared publicly because of [XXX]. Data are available from the XXX Institutional Data Access / Ethics Committee (contact via XXX) for researchers who meet the criteria for access to confidential data.* | All relevant data are within the manuscript and its Supporting and Information files. |

| | |
|---|---|
| *The data underlying the results presented in the study are available from (include the name of the third party and contact information or URL).*<br>• This text is appropriate if the data are owned by a third party and authors do not have permission to share the data.<br><br>* typeset | |
| Additional data availability information: | |

Powered by Editorial Manager® and ProduXion Manager® from Aries Systems Corporation

Manuscript

Click here to access/download;Manuscript;Revised Manuscript without Track Changes.docx

RESEARCH ARTICLE

# Willingness to work in rural areas and associated factors among graduating medical and health science students at the University of Gondar, northwest Ethiopia, 2021

**Blen Getachew[1], Biruk Bizuneh[1], Birhanu Ewunetu[1], Dawit Kassahun[1], Dessalegn Fentahun[1], Destaw Ademe[1], Endeshaw Admasu Cherkos[2], Azmeraw Ambachew Kebede[3*]**

[1] Department of General Midwifery, College of Medicine and Health Sciences, University of Gondar, Gondar Ethiopia; [2] Department of Women's and Family Health, School of Midwifery, College of Medicine and Health Sciences, University of Gondar, Gondar, Ethiopia; [3] Department of Clinical Midwifery, School of Midwifery, College of Medicine and Health Sciences, University of Gondar, Gondar, Ethiopia

[*] azmuzwagholic@gmail.com

# Abstract

## Background

Many of the rural areas in developing countries are still in need access to quality healthcare services. To ensure the fair distribution of a high-quality health workforce and the availability of health services, there is a need to assess the background barriers that explain why healthcare providers are not interested to work in rural areas, thereby setting strategies to achieve universal health coverage. Therefore, this study is aimed to assess the willingness to work in rural areas and associated factors among medical and health science students at the University of Gondar.

## Methods

An institution-based cross-sectional study was conducted at the University of Gondar from August 15 to 25, 2021. A total of 422 study participants were selected using a stratified random sampling technique. A pretested self-administered questionnaire was employed to collect the data. Data were entered into EPI DATA 4.6 and exported to SPSS 25 for further analysis. Multivariable logistic regression analysis was performed to identify factors associated with students' willingness to work in rural areas. The level of significance was decided based on the 95 % confidence interval at a p-value of $\leq 0.05$.

## Results

In this study, it was found that medical and health science students' willingness to work in rural areas was 78.4% (95% CI: 74.3, 82.4). Being male (AOR = 2.15; 95% CI: 1.17, 3.94), having intention to continue with their profession (AOR = 2.5; 95% CI: 1.28, 4.86), having a favorable attitude towards working in rural areas (AOR = 7.32; 95% CI: 5.71, 18.65), and having a mother with no formal education (AOR = 2.23; 95% CI: 1.02, 4.85) and completed primary education (AOR = 2.69; 95% CI: 1.1, 6.61) were significantly associated with willingness to work in rural areas.

## Conclusion

The willingness of students to work in rural areas was optimal. This calls for concerned bodies to create a conducive environment for male and female students to engage in rural areas without

hesitation. It is also important to ensure that students are willing to work in the rural areas voluntarily, instead of working in rural areas due to a lack of employment.

Keywords: Ethiopia, graduating students, medical and health sciences, rural health services, willingness

# Introduction

The health workforce plays a key role in the provision of health. The equitable distribution of quality health personnel contributes significantly to ensuring the availability of healthcare services, irrespective of location. Besides, it helps in progressing toward the Universal Health Coverage (UHC) goal by facilitating access to quality healthcare services for all [1].

More than 4 million health workers are needed to fill the gap in the global health workforce, as estimated by the  World Health Organization (WHO) [2]. However, the geographical distribution of health workers is concentrated in affluent cities and regions worldwide, paying little attention to the rural areas. Regardless of the level of economic development and organization of the health system, the disparity in the distribution of health workers exists in almost every country in the world, but this problem is particularly prominent in developing countries [3].

The shortage of health workers in rural areas has been identified as one of the biggest challenges for most countries to achieve the desired health-related goals. Developing countries suffer severely due to a significant shortage of health workers proportional to the population [4]. Ethiopia is also one of the victims of this challenge, despite 84% of its citizen residing in rural areas [5]. One of the challenges to provide at least basic health services is the lack of trained health workers [6]. In connection with this, the WHO recommends that at least 4.45 doctors, nurses, and midwives per 1000 population are needed to achieve the minimum levels of key health interventions. However, Africa has a 2.2 health workforce per 1000 population, which is often challenging to achieve global goals [1].

In Ethiopia, the overall health professionals to population ratio were 1 per 1,000 population in 2018 [7], which is far below the minimum number estimated by the WHO. Hence, the healthcare system still suffers from the shortage mentioned earlier and the uneven distribution of healthcare workers between the urban and rural populations [8]. The shortage of health staff also has a significant impact on the healthcare delivery system such as the provision of essential life-saving

interventions such as providing Expanded Program on Immunization (EPI), clean water, safe pregnancy, and childbirth services, and access to Human Immunodeficiency deficiency Virus/Acquired Immunodeficiency Syndrome (HIV/AIDS), tuberculosis (TB), and malaria treatment [9].

The Sustainable Development Goals (SDG) by United Nations (UN) member states in 2015, particularly goal 3 aim to ensure health and wellbeing for all. Target 3.1 specifically aims to reduce the Maternal Mortality Ratio (MMR) to less than 70 per 100,000, thereby saving the lives of one million mothers [10]. In addition, 405,000 people died of malaria in 2018, 94% of which occurred in Africa [11]. Moreover, an estimated 1.3 million TB-related deaths were recorded in 2017; additional 300,000 deaths among people living with HIV/AIDS [12]. All these burdens are more common in rural areas. Shortage and mal-distribution of healthcare workers, particularly in rural areas will lead to failure to meet the SDGs. Hence, assigning adequate healthcare providers to these highly vulnerable groups will greatly help reduce the deaths of many lives.

Higher prevalence of mortality and morbidity are found in rural areas compared with urban areas [13]. Thus, the willingness of healthcare providers to work in rural areas is crucial to basically alleviating the problem. However, most health care providers are reluctant to work in rural settings. Some of the commonly cited reasons for not working in these areas are perceived low salary, unavailability of professional standards, unsatisfactory working environment, lack of access to skilled colleagues, and modern equipment [9]. Besides, some others stated that learning and training opportunities are scarce when they get employed in rural areas [14]. Furthermore, lack of the expected living conditions including electricity, water supply, and schools for their children, and having a plan to migrate out of their country after graduation are some of the reasons that lower the willingness to work in rural areas [14,15]. Thus, it is important to give adequate remuneration to their work for health workers, better professional training, and educational opportunities, and identify effective and efficient human and non-human resources to meet the individual health needs of the underserved rural population.

Medical and health science students' desire to go to the countryside will play an important role in the rural community that has been denied access to health services. Studies so far revealed that students willingness to work in rural areas were 52% in China [16], 45% in India [17], 33.5% in Nigeria [18], 55.4% in Ghana [19], and 29.7% in Ethiopia [20]. Being a male student [16,19],

having a family member of lower academic attainments and lower socioeconomic status [21], rural background [22], and having a good attitude towards the rural community [15] had a positive impact on students' willingness to work in the rural areas.

Although the health workforce in Ethiopia is growing, it is still less than half of the minimum number estimated by WHO. Increasing the willingness of graduating students by designing effective strategies will play a key role in reducing the burden of shortage of health care workers in the rural areas, thereby achieving the optimal health of the marginalized rural population. Besides, doing the research may offer basic evidence aimed at improving health workers' needs and setting strategies considering the rural community. Therefore, this study aimed to assess willingness to work in rural areas and associated factors among graduating medicine and health science students at the University of Gondar, northwest Ethiopia.

# Methods and materials

## Study design, period, and study area

An institution-based cross-sectional study was conducted from August 1 to 25, 2021. The study was conducted at the University of Gondar, College of Medicine and Health Sciences, which is found in Gondar city, northwestern Ethiopia. The college was established in 1954 and is considered one of the oldest and most famous Universities in Ethiopia. There are 12 fields of study in health and medicine such as Nursing, Midwifery, Clinical laboratory, Pharmacy, Anesthesia, Psychiatry, Health informatics, Physiotherapy, Optometry, Environmental and occupational health, Public health officer (HO), and Medicine. Currently, there are 1068 graduating students from the 12 departments by September 2021.

## Study population

All graduating medical and health science students during the study period were the study population.

## Sample size determination and sampling procedure

The sample size for this study was calculated based on the assumptions of the single population proportion formula by considering the following assumptions. Since there is no similar study, we

used the proportion of student's willingness to work in the rural area-50% (p = 0.5), level of significance- 5 % (α = 0.05), Z α/2 - 1.96, and margin of error - 5% (d = 0.05). Therefore, the sample size was calculated as follows

$$n = \frac{(Z\alpha/2)^2 * p(1-p)}{d^2} = \quad n = \frac{(1.96)^2 * 0.5(1-0.5)}{(0.05)2} = 384.$$

After adding a 10% non-response rate, the minimum adequate sample size was found to be 422. There are 12 departments at the University of Gondar, college of medicine, and health sciences. All departments were included in the study. The lists of students were obtained from the registrar's office and the sampling frame was prepared by ordering the lists of students. Then, the total sample size was distributed proportionally to each department. Finally, the participants were selected using a stratified random sampling technique using a table of random generation **(Figure 1).**

## Variables of the study

The willingness of students to work in the rural areas was the outcome variable, whereas age, sex, place of origin, religion, professional category, paternal and maternal educational status, paternal and maternal occupation, and personal behaviors like exposure to addictive substances, attitude to work in the rural area, and plan to emigrate were the independent variables.

## Measurements

**Willingness to work in rural areas:** Students were asked whether they are willing to work in rural areas or not. A "yes" response was considered as willing to work in the rural areas [19].

**Favorable attitude towards working in rural areas**: Students' attitude toward working in rural areas was assessed using 11 questions: 1) Working in rural areas provide opportunities to use various skills 2) There is a supportive environment when working in a rural environment 3) Working in rural areas limits communication with professional peers 4) Working in rural areas provide opportunities to work independently 5) There is lack of amenities and entertainment in rural areas 6) People in rural areas are friendly 7) Working in these areas causes isolation from family and friends 8) Working as a health care provider in hospitals or health centers in rural areas is the most important contribution to the health of the population 9) Health science college prepared me well to work in rural areas 10) Working in rural hospitals areas is the most

challenging 11) Working in rural hospitals provide opportunities for real-life problem-solving. Each question has five points Likert scale (1- strongly disagree, 2- disagree, 3- neutral, 4- agree, and 5- strongly agree). The minimum and maximum scores were 11 and 55, respectively. Thus, students who answered above the mean value are considered as having a favorable attitude [17].

## Data collection tools and procedures

The data collection tool was developed by reviewing the literature [16,17,19,20] and data were collected using a structured, pretested, and self-administered questionnaire. The questionnaire was evaluated by experts before data collection. The questionnaire contains socio-demographic characteristics and academic-related characteristics. Six BSc in midwifery graduating students and 1 MSc in Midwifery holder were recruited for data collection and supervision, respectively.

## Data quality controls

To ensure consistency and understandability, the questionnaire was initially prepared in English and translated to the local Amharic language and back to English. A pretest was done on 5% of the calculated sample size at Bahir Dar University to check the response, language intelligibility, and relevance of the questionnaire. The data collectors and supervisors were trained for a day regarding the overall data collection process. At the time of actual data collection, the collected data was checked daily by the supervisor for completeness.

## Data processing and analysis

Data were entered into EPI DATA 4.6 and exported to SPSS version 25 for further cleaning and analysis. Tables, figures, mean, and proportions are used to state the descriptive statistics of the study participants. The chi-square assumption was tested. Binary logistic regression was fitted to identify independent predictors. Variables with a p-value of less than 0.25 in the bivariable logistic regression were included in the multivariable logistic regression to identify the final independent predictors. Hosmer-Lemeshow was used to test the model's goodness of fit. In addition, variance inflation factor (VIF), and standard error were used to screen multi-collinearity among independent variables. In the multivariable logistic regression analysis, a p-value of $\leq 0.05$ and 95% CI for the adjusted odds ratio (AOR) were used to determine the

significance and degree of association between willingness to work in rural areas and the explanatory variables.

## Ethical considerations

The ethical clearance letter was obtained from the School of Midwifery under the delegation of the Institutional Review Board (IRB) of the University of Gondar. A formal administrative approval letter was gained from the college of medicine and health sciences. After clearly explaining the purpose of the study to the students, anonymous written informed consent was obtained from each study participant. Interviewees were given the right to refuse and withdraw from the study at any time.

# Result

## Socio-demographic characteristics of study participants

A total of 422 study participants were recruited but only 402 of them completed the study, giving a response rate of 95.26%. The mean age (standard deviation) of the students was 23.9 years old (± 2.28) and the majority (70.4%) of students were in the age group of < 25 years. Nearly two-thirds (62.4%) of the study participants were males. About 29.4% of the participants were medical students and 46% of them were from urban areas by birthplace **(Table 1).**

## Academic, medical, and behavior-related characteristics

Of the total study participants, nearly three-fourths (73.9%) believed that more job opportunities are available in urban areas. About 58.5% of the study participants joined their first departmental choice and got information regarding the department before campus entry. More than two-thirds (68.4%) and fourth-fifths (81.1%) of the study participants had a favorable attitude towards working in rural areas and intended to stay in their profession, respectively **(Table 2).**

## Willingness to work in rural areas and associated factors among graduating students

The willingness of students to work in rural areas was 78.4% (95% CI: 74.3, 82.4). In the bivariable logistic regression, sex, age, mother's educational status, father's educational status,

place of birth, intention to continue with the profession, and attitude towards working in the rural areas were associated with willingness to work in rural areas. However, in the multivariable logistic regression analysis being male, intention to continue with the profession, having a mother with no formal education and attended primary education, and having a favorable attitude towards working in rural areas were variables significantly associated with willingness to work in rural areas.

Hence, the odds of having a good willingness to work in rural areas were two times higher among male students (AOR = 2.15; 95% CI: 1.17, 3.94) as compared to female students. Similarly, students who had an intention to continue with their profession were 2.5 times higher (AOR = 2.5; 95% CI: 1.28, 4.86) compared with those students who had no intention. Likewise, the likelihood of having a willingness to work in rural areas was 7.32 times higher (AOR = 7.32; 95% CI: 5.71, 18.65) among students who had a favorable attitude towards working in rural areas as compared to their counterparts. Lastly, students who had a mother with no formal education and attended primary education were 2.23 (AOR = 2.23; 95% CI: 1.02, 4.85) and 2.69 times (AOR = 2.69; 95% CI: 1.1, 6.61) more likely to have had a willingness to work in rural areas as compared to students who had a mother attended college and above education, respectively (**Table 3**).

## Perceived reasons for not willing to work in rural areas

About 21.6% (87) of the study participants were not willing to work in rural areas after graduation. The frequently mentioned reasons by the students for not to work in rural areas include lack of infrastructure in rural areas, not being far from family members, and peace and security issues **(Figure 2).**

# Discussion

This study aimed to assess the willingness to work in rural areas, and associated factors among graduating medical and health science students at the University of Gondar, northwest Ethiopia.

This study found that student's willingness to work in rural areas was 78.4%, which was far higher than studies conducted in Egypt-52.3% [14], China-52% [16], India-45% [17], Nigeria-33.5% [18], Ghana-55.4% [19], and Ethiopia-29.7% [20]. The possible explanation for the

variation might be differences in socio-demographic contexts and time gaps. More than 80% of Ethiopia's population lives in rural areas [5], which may need more health workers, and new job opportunities in the health care system are available there. Besides, the number of health workers might be limited so far, and jobs might be available in the urban areas. Nowadays, however, there might be a higher number of health professionals than ever before, and jobs may not be easy to find, especially in Ethiopia. So newly graduating students may want to work in rural areas unreservedly. In light of this, the Ethiopian government and other stakeholders should address and implement conditions for job creation for medical and health science students, and arrange opportunities that will favor students to work in rural areas in all aspects. In turn, this idea will reduce the student’s intention to leave their profession and burnout in the workplace.

Regarding factors associated with willingness to work in rural areas, this study affirmed that the sex of students affects their willingness to work in rural areas. Accordingly, male students were two times more likely to have had a good willingness to work in rural areas as compared to their female counterparts. This finding is consistent with studies conducted in Egypt and Ghana [16,19]. This can be explained by the fact that male students might cope with problems that are considered to be present in rural areas, such as transportation problems, lack of electricity, and lack of safe water. When there is a lack of transportation, health workers may walk to the city on foot. Thus, females may be afraid that they will be in trouble. However, this finding contradicts a study conducted in China that founds female students intend to work more in rural areas compared with their male counterparts [21]. This could be justified by differences in the country’s infrastructure and culture of the community. In Ethiopia, the infrastructure might not be favorable for females like that of China. Besides, female students might not be willing to be far from families because of the perception of the community, which the community may not believe females can deal with the difficult environment.

This study revealed that students who had a mother with no formal education and had attended primary education were 2.23 and 2.69 times more likely to have had a willingness to work in rural areas as compared to students who had a mother who had attended college and above education, respectively. This finding is in agreement with a study conducted in China [21]. This could be explained by parents who might have a big influence on shaping and directing the attitude and behaviors of their children, including choosing departments and workplaces.

Besides, those students who had a family with less educational achievement are expected to be less affluent. Thus, to support the household economy, students will have a willingness to work in rural areas irrespective of preconditions.

In this study, the odds of willingness to work in rural areas were two times higher among students who had plans to continue with their profession as compared to those students who had no intention to continue with the profession that they have currently. A study conducted in Nigeria reported a similar result [23]. This could be explained by the fact that students who intend to continue with their profession may initially join the department of their choice and could have a strong commitment to work in any part of the country. On the other hand, students may not have the interest to continue with their current profession due to different reasons like a plan to immigrate to foreign counties and engage in other business. Because those students with no intention to continue with their profession might have a plan to participate in other income-generating activities and plan to change their profession due to a lack of interest in the department after they are engaged. In this regard, the Ethiopian government should pay attention to the healthcare providers by settling different encouraging undertakings such as crafting job opportunities, evenhanded salary for their work, and timely educational opportunities for their vertical growth.

This study also found that students' attitude toward rural work affected their willingness to work in rural areas after graduation. Accordingly, students with a positive attitude towards working in rural areas were seven times more likely than students with unfavorable attitudes. This result is consistent with a study conducted in Ethiopia [15]. The attitude of an individual has a direct impact on determining responses to situations, psychological preparations, and influencing responses to all situations around them [17]. Thus, students who have a positive attitude to the need for rural community support and access to equitable health care may want to work in rural areas unconditionally. They might also be happy to help people, and able to fulfill their good wishes by serving the rural people, where highly in need of healthcare access. Lastly, we are pleased to acknowledge some of the limitations of this study. Due to the cross-sectional nature of the study design, it may not be possible to know the exact effect of the identified factors on the outcome variable. Moreover, since the questionnaire was self-administered, response bias might be introduced. However, before data collection, students were told that their participation in the

research is significant and that they would fill in the required information honestly for the success of the research.

# Conclusion

More than three-quarters of medical and health science students were willing to work in rural areas. Being a male student, having the intention to stay in their profession, having a good attitude toward working in rural areas, and students having a mother of low academic achievement were observed to affect the student's willingness to work in the rural areas. Thus, this calls for health policymakers and other stakeholders to give attention to arranging all-encompassing job opportunities in the rural areas that will favor both males and females. In addition, it is important to build a good view and understanding for students about rural areas during their campus life.

## Supporting information

S1 Questionnaire: English and Amharic version.

S2 Dataset: SPSS data.

# Authors' Contributions

**Conceptualization**: Azmeraw Ambachew Kebede

**Data curation**: Blen Getachew, Biruk Bizuneh, Birhanu Ewunetu, Dawit Kassahun, Dessalegn Fentahun, Destaw Ademe, Endeshaw Admasu Cherkos, Azmeraw Ambachew Kebede

**Formal analysis:** Blen Getachew, Biruk Bizuneh, Birhanu Ewunetu, Dawit Kassahun, Dessalegn Fentahun, Destaw Ademe, Endeshaw Admasu Cherkos, Azmeraw Ambachew Kebede

**Investigation:** Blen Getachew, Biruk Bizuneh, Birhanu Ewunetu, Dawit Kassahun, Dessalegn Fentahun, Destaw Ademe, Endeshaw Admasu Cherkos, Azmeraw Ambachew Kebede

**Methodology:** Blen Getachew, Biruk Bizuneh, Birhanu Ewunetu, Dawit Kassahun, Dessalegn Fentahun, Destaw Ademe, Endeshaw Admasu Cherkos, Azmeraw Ambachew Kebede

**Validation:** Blen Getachew, Biruk Bizuneh, Birhanu Ewunetu, Dawit Kassahun, Dessalegn Fentahun, Destaw Ademe, Endeshaw Admasu Cherkos, Azmeraw Ambachew Kebede

**Visualization:** Blen Getachew, Biruk Bizuneh, Birhanu Ewunetu, Dawit Kassahun, Dessalegn Fentahun, Destaw Ademe, Endeshaw Admasu Cherkos, Azmeraw Ambachew Kebede

**Writing-original draft:** Blen Getachew and Azmeraw Ambachew Kebede

**Writing- review and editing:** Blen Getachew, Biruk Bizuneh, Birhanu Ewunetu, Dawit Kassahun, Dessalegn Fentahun, Destaw Ademe, Endeshaw Admasu Cherkos, Azmeraw Ambachew Kebede

# Acknowledgments

We would like to thank the University of Gondar for providing study ethical clearance to conduct this study. Our gratitude also goes to all data collectors and study participants. We are glad to Hospitals in Gondar province for writing permission letter.

**Data Availability Statement**

All relevant data are within the manuscript and its Supporting information files.

**Funding:** The author(s) received no specific funds for this work.

**Competing interests**: The authors declare that they have no conflict of interest in this work

**Abbreviations**

AOR; Adjusted Odds Ratio, CI; Confidence Interval, COR Crude Odds Ratio, SPSS; Statistical Package for Social Science, SSA; Sub-Saharan Africa, WHO; World Health Organization

Table 1: Socio-demographic characteristics of study participants in the University of Gondar, college of medicine and health sciences, northwest Ethiopia, 2021 (n = 402)

| Charactestics | Frequency | Percentage (%) |
|---|---|---|
| **Age in year** | | |
| <25 | 283 | 70.4 |
| ≥25 | 119 | 29.4 |
| **Sex** | 251 | 62.4 |
| Male | 151 | 37.6 |

| | | |
|---|---|---|
| Female | | |
| **Place of birth** | | |
| Urban | 185 | 46 |
| Semi-urban | 59 | 14.7 |
| Rural | 158 | 39.3 |
| **Religion** | | |
| Orthodox Christian | 344 | 85.6 |
| Protestant | 42 | 10.4 |
| Others[a] | 16 | 4 |
| **Current marital status** | | |
| Married | 19 | 4.7 |
| In relationship | 31 | 7.7 |
| Single | 352 | 87.6 |
| **Average monthly pocket money** | | |
| < 500 ETB | 70 | 17.4 |
| 500-999 ETB | 108 | 26.7 |
| ≥ 1000 ETB | 224 | 55.7 |
| **Department** | | |
| Medicine | 118 | 29.4 |
| Nursing | 43 | 10.7 |
| Midwifery | 33 | 8.2 |
| Public health officer | 32 | 8 |
| Psychiatry | 17 | 4.2 |

| | | |
|---|---|---|
| Environmental and occupational health | 21 | 5.2 |
| Optometry | 17 | 4.2 |
| Physiotherapy | 17 | 4.2 |
| Health informatics | 20 | 5 |
| Anesthesia | 18 | 4.5 |
| Pharmacy | 35 | 8.7 |
| Medical laboratory | 31 | 7.7 |
| **Average monthly income of the family** | | |
| <1500 ETB | 14 | 3.5 |
| 1501-5000 ETB | 115 | 28.6 |
| ≥ 5001 ETB | 273 | 67.9 |
| **Mothers educational status** | | |
| No formal education | 159 | 39.6 |
| Primary education | 83 | 20.6 |
| Secondary education | 67 | 16.7 |
| Diploma and above | 93 | 23.1 |
| **Mother's occupation** | | |
| Housewife and/or farmer | 229 | 57 |
| Government employee | 67 | 16.7 |
| Self employed | 47 | 11.7 |
| Merchant | 57 | 14.2 |
| Others[b] | 2 | 0.5 |

| | | |
|---|---|---|
| **Father's educational status** | | |
| No formal education | 110 | 27.4 |
| Primary | 120 | 29.9 |
| Secondary | 47 | 11.7 |
| Diploma and above | 125 | 31.1 |
| **Fathers occupation** | | |
| Farmer | 168 | 41.8 |
| Merchant | 74 | 18.4 |
| Government employed | 105 | 26.1 |
| Self employed | 53 | 13.2 |
| Others[b] | 2 | 0.5 |

a = Muslim and catholic, b = retired, ETB- Ethiopian Birr

Table 2: Academic, medical, and behavioral related characteristics of study participants in the University of Gondar, college of medicine and health sciences, northwest Ethiopia, 2021 (n = 402)

| Charactestics | Frequency | Percentage (%) |
|---|---|---|
| **History of khat chewing** | | |
| Yes | 33 | 91.8 |
| No | 369 | 8.2 |
| **History of cigarette smoking** | | |
| Yes | 23 | 5.7 |
| No | 379 | 94.3 |
| **Ever drunk alcohol** | | |
| Yes | 178 | 55.7 |

| | | |
|---|---|---|
| No | 224 | 44.3 |
| **Getting information about the department before campus entry** | | |
| Yes | 240 | 59.7 |
| No | 162 | 40.3 |
| **Source of information (n = 240)** | | |
| Friends | 116 | 48.3 |
| Healthcare providers | 17 | 7.1 |
| Families | 61 | 25.4 |
| Mass media | 46 | 19.2 |
| **Is the department your first choice** | | |
| Yes | 235 | 58.5 |
| No | 167 | 41.5 |
| **Intention to continue with the profession** | | |
| Yes | 329 | 81.1 |
| No | 73 | 18.2 |
| **Attitude towards working in rural areas** | | |
| Favorable | 275 | 68.4 |
| Unfavorable | 126 | 31.1 |
| **Where did you think jobs are available** | | |
| Urban | 297 | 73.9 |
| Rural | 105 | 26.1 |
| **Do you have any known medical disorder** | | |

| | | |
|---|---|---|
| Yes | 384 | 95.5 |
| No | 18 | 4.5 |

Table 3: Bivariable and multivariable logistic regression analysis of factors associated with graduating students willingness to work in rural areas in the University of Gondar, northwest Ethiopia, 2021 (n = 402)

| Variables | Willingness to work in rural areas | | COR ( 95% CI) | AOR ( 95% CI) |
|---|---|---|---|---|
| | Yes | No | | |
| **Sex** | | | | |
| Male | 214 | 37 | 2.86 (1.76, 4.65) | **2.15 (1.17, 3.94)*** |
| Female | 101 | 50 | 1 | 1 |
| **Age in year** | | | | |
| < 25 | 213 | 70 | 1 | 1 |
| ≥ 25 | 102 | 17 | 1.97 (1.10, 3.52) | 1.35 (0.66, 2.75) |
| **Place of birth** | | | | |
| Urban | 132 | 53 | 1 | 1 |
| Semi-urban | 46 | 13 | 1.42 (0.71, 2.84) | 0.92 (0.36, 2.31) |
| Rural | 137 | 21 | 2.62 (1.49, 4.58) | 1.61 (0.63, 4.12) |
| **Intention to continue with the profession** | | | | |
| Yes | 267 | 62 | 2.24 (1.28, 3.91) | **2.50 (1.28, 4.86)*** |
| No | 48 | 25 | 1 | 1 |
| **Mother's educational status** | | | | |
| No formal education | 137 | 22 | 2.54 (1.35,4.81) | **2.23 (1.02, 4.85)*** |
| Primary education | 72 | 11 | 2.68 (1.23, 5.82) | **2.69 (1.1, 6.61)*** |

| | | | | |
|---|---|---|---|---|
| Secondary education | 40 | 27 | 0.6 (0.31, 1.17) | 0.58 (0.268, 1.27) |
| Diploma and above | 66 | 27 | 1 | 1 |
| **Father's educational status** | | | | |
| No formal education | 96 | 14 | 3.35 (1.70, 6.56) | 3.35 (1.00, 10.36) |
| Primary education | 102 | 18 | 2.76 (1.48, 5.16) | 1.62 (0.66, 4.01) |
| Secondary education | 33 | 14 | 1.15 (0.55, 2.38) | 1.03 (0.39, 2.72) |
| Diploma and above | 84 | 41 | 1 | 1 |
| **Attitude towards working in the rural area** | | | | |
| Favorable | 249 | 26 | 8.7 (5.10, 14.85) | **7.32 (5.71,18.65)**** |
| Unfavorable | 66 | 60 | | 1 |

Notes: * P ≤ 0.05, **P ≤ 0.001

Abbreviations: AOR, adjusted odds ratio; COR, crude odds ratio; CI, confidence interval; 1, reference category

**Reference**


1. World Health Organization. (2016). Health workforce requirements for universal health coverage and the Sustainable Development Goals. (Human Resources for Health Observer, 17). World Health Organization. https://apps.who.int/iris/handle/10665/250330.

2. World Health Organization. (2006). The world health report: 2006: Working Together for Health. World Health Organization. Available from: https://apps.whoint/iris.handle/10665/43432

3. Asante AD, Zwi AB. Factors influencing resource allocation decisions and equity in the health system of Ghana. 2009;123(5):371-377. DOI: https://doi.org/10.1016/j.puhe.2009.

4. Liu JX, Goryakin Y, Maeda A, Bruckner T, Scheffler R. Global Health Workforce Labor Market Projections for 2030. Hum Resour Health. 2017;15(1. DOI: 10.1186/s12960-017-0187–2).

5. Assefa Y, Hill PS, Gilks CF, Admassu M, Tesfaye D, Van Damme W. Primary health care contributions to universal health coverage, Ethiopia. Bull World Health Organ. 2020;98(12.):894-905.DOI: 10.2471/BLT.19.248328.

6. Mengistu BS, Vins H, Kelly CM, McGee DR, Spicer JO, Derbew M, et al. Student and faculty perceptions on the rapid scale-up of medical students in Ethiopia. BMC Med Educ. 2017;17(1):1-8. DOI:10.1186/s12909-016-0849–0.

7. Health Sector Transformation Plan II 2020/2021-2024/2025. Ethiop Minist Heal [Internet]. 2021;25:1–128. Available from: e-library.moh.gov.et/library/index/php/knoledge-product/health -sector-transformation-plan-ii-sstp-ii-2020-21-2024-25/

8. Bradley E, Thompson JW, Byam P, Webster TR, Zerihun A, Alpern R, et al. Access and quality of rural healthcare: Ethiopian Millennium Rural Initiative. Int J Qual Heal Care. 2011;23(3):222-230. DOI:10.1093/intqhc/mzr013.

9. Girasek E, Eke E, Szócska M. Analysis of a survey on young doctors' willingness to work in rural Hungary. Hum Resour Health. 2010;8(1):1-6. DOI: 10.1186/1478-4491-8–13.

10. SDG. Final list of proposed Sustainable Development Goal indicators. Report of the Inter-

Agency and Expert Group on Sustainable Development Goal Indicators (E/CN.3/2016/2/Rev.1). 2016; Available from: https://sustainabledevelopment.un.org/content/documents/11803Official-List-of-Proposed-SDG-Indicators.pdf

11. Regional and global trends in burden of malaria cases and deaths. World Health Organization malaria report 2019. Available from: https://www.who.int/news-room/feature-stories/detail/world-malaria-report-2019.

12. Global tuberculosis report 2018. Geneva: World Health Organization; 2018. Licence: CC BY-NC-SA 3.0 IGO.

13. Curtin SC, Spenncer MR. Trends in death rates in urban and rural areas: United States, 1990-2019. NCHS Data Brief, no 417. Hyattsville, MD: National Center for Health Statistics. 2021. DOI: https://dx.doi.10.15620/cdc.109049.

14. Mohamed AM. Willingness and professional motivations of medical students to work in rural areas: a study in Alexandria, Egypt. Healthc Low-resource Settings. 2013;1(1):DOI: 10.4081/HLS.2013.E4.

15. Deressa W, Azazh A. Attitudes of undergraduate medical students of Addis Ababa University towards medical practice and migration, Ethiopia. BMC Med Educ. 2012;12(1):DOI: 10.1186/1472-6920-12-68.

16. Liu J, Zhu B, Mao Y. Association between rural clinical clerkship and medical students' intentions to choose rural medical work after graduation: A cross-sectional study in western China. PLoS One. 2018;13(4):DOI: 10.1371/JOURNAL.PONE.0195266.

17. Rajesh Kumar Singh, CMS Rawat SP. Attitude of medical students toward serving in rural areas and its determinants: a cross-sectional study from Uttarakhand. Int J Med Sci Public Heal. 2015;4(6):8-14.DOI: 10.5455/ijmsph.2015.03032015166.

18. Ndu AC, Aniwada EC, Umeh EN, Uzo NM, Uzoma CE. Perception and willingness of medical students towards working in rural communities. Niger J Med. 2019;28(3):275-280. DOI:10.4314/njm.v28i3.

19. Agyei-Baffour P, Kotha SR, Johnson JC, Gyakobo M, Asabir K, Kwansah J, et al.

Willingness to work in rural areas and the role of intrinsic versus extrinsic professional motivations - A survey of medical students in Ghana. BMC Med Educ. 2011;11(1):1-8. DOI: 10.1186/1472-6920-11–56.

20. Assefa T, Haile Mariam D, Mekonnen W, Derbew M. Medical students' career choices, preference for placement, and attitudes towards the role of medical instruction in Ethiopia. BMC Med Educ. 2017;17(1):1-10. DOI: 10.1186/s12909-017-0934-z.

21. Liu J, Zhang K, Mao Y. Attitude towards working in rural areas: a cross-sectional survey of rural-oriented tuition-waived medical students in Shaanxi, China. BMC Med Educ 2018 181. 2018;18(1):1-11. DOI: 10.1186/S12909-018-1209-Z.

22. Serneels P, Montalvo JG, Pettersson G, Lievens T, Buterae JD, Kidanu A. Who wants to work in a rural health post? The role of intrinsic motivation, rural background and faith-based institutions in Ethiopia and Rwanda. Bull World Health Organ. 2010;88(5):342-349. DOI: 10.2471/BLT.09.072728.

23. E N Ossanai, A F Una, R C Onyenakazi EUN. Emigration Plans after Graduation of Clinical Medical Students of Ebonyi State University Abakaliki, Nigeria: Implications for Policy. Niger J Clin Pract. 2020;23(7):995-1003. DOI: 10.4103/njcp.njcp.

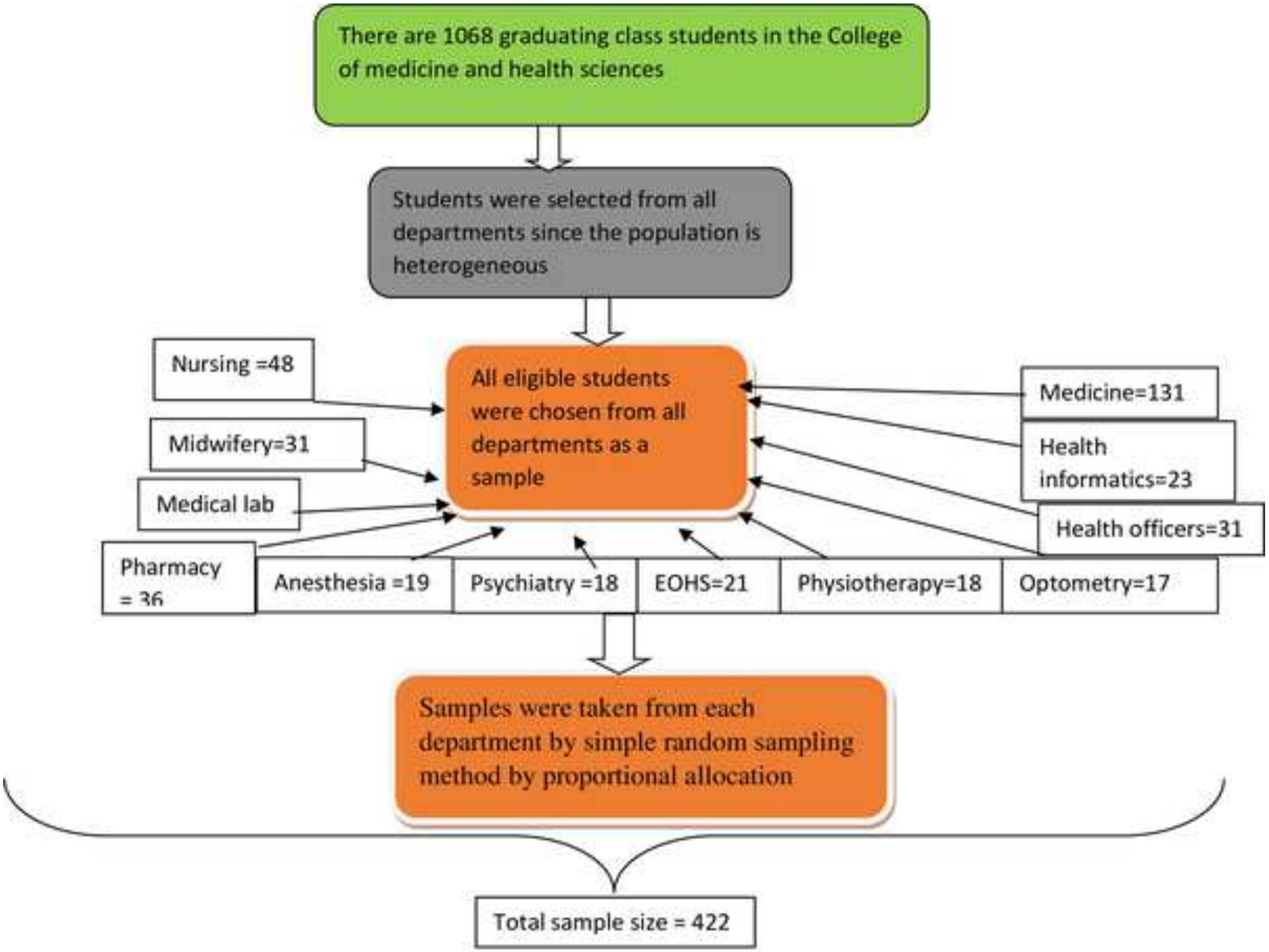


Figure 1: Schematic presentation of the sampling procedure for the study done on willingness to work in rural areas and associated factors among graduating medicine and health science students at university of Gondar, 2021

Click here to access/download;Figure;Figure 2.tif

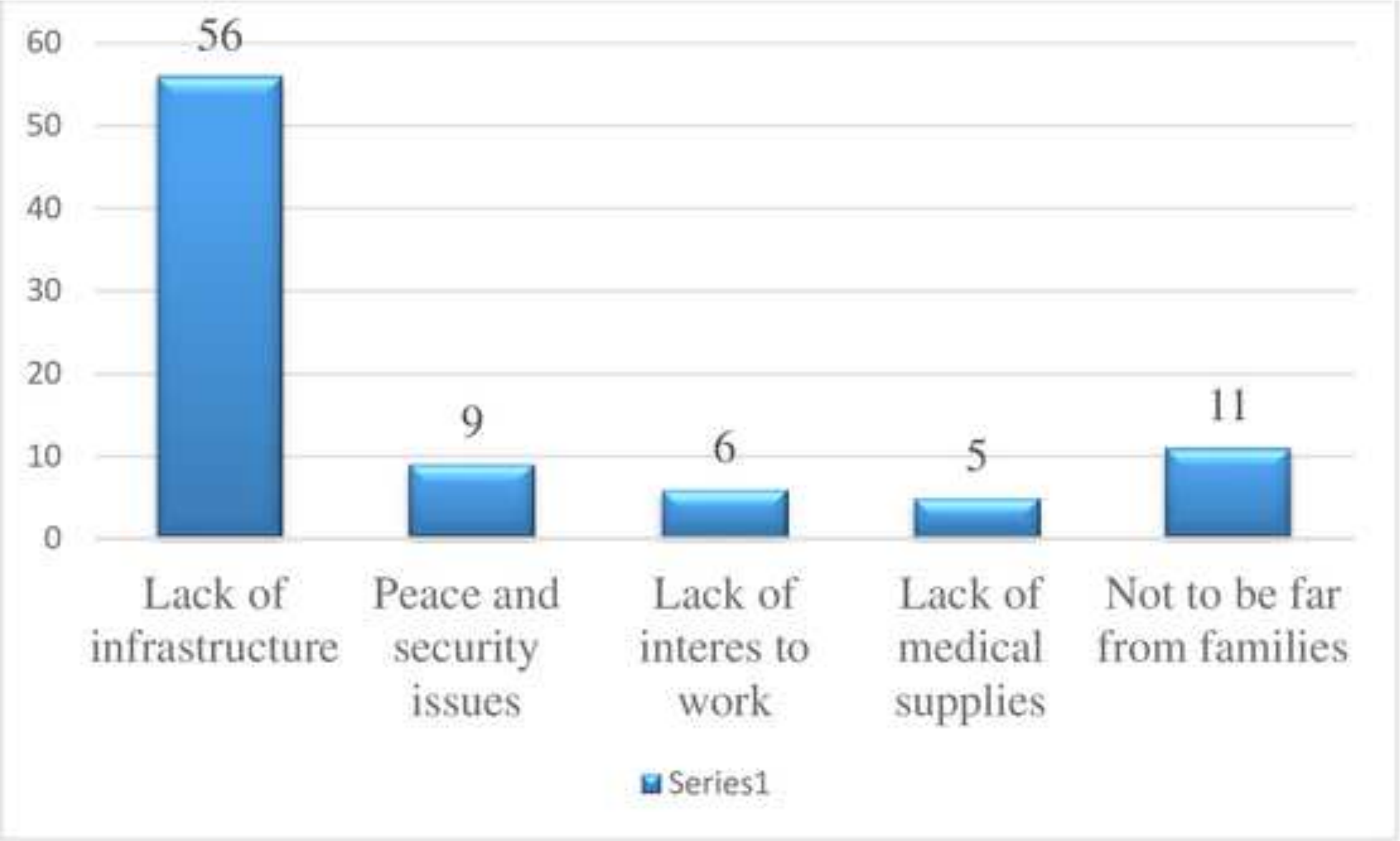


Figure 2: Reasons why students are unwilling to work rural in rural areas at the University of Gondar, northwest Ethiopia, 2021.

Questionnaire

Click here to access/download
**Supporting Information**
English and amhraric version of questionnaire.docx

Questionnaire

Click here to access/download
**Supporting Information**
SPSS data.sav

RESEARCH ARTICLE

# Willingness to work in rural areas and associated factors among graduating medical and health science students at the University of Gondar, northwest Ethiopia, 2021

**Blen Getachew[1], Biruk Bizuneh[1], Birhanu Ewunetu[1], Dawit Kassahun[1], Dessalegn Fentahun[1], Destaw Ademe[1], Endeshaw Admasu Cherkos[2], Azmeraw Ambachew Kebede[3*]**

[1] Department of General Midwifery, College of Medicine and Health Sciences, University of Gondar, Gondar Ethiopia; [2] Department of Women's and Family Health, School of Midwifery, College of Medicine and Health Sciences, University of Gondar, Gondar, Ethiopia; [3] Department of Clinical Midwifery, School of Midwifery, College of Medicine and Health Sciences, University of Gondar, Gondar, Ethiopia

[*] azmuzwagholic@gmail.com

# Abstract

## Background

Many of the rural areas in developing countries are still in need access to quality healthcare services. To ensure the fair distribution of a high-quality health workforce and the availability of health services, there is a need to assess the background barriers that explain why healthcare providers are not interested to work in rural areas, thereby setting strategies to achieve universal health coverage. Therefore, this study is aimed to assess the willingness to work in rural areas and associated factors among medical and health science students at the University of Gondar.

## Methods

An institution-based cross-sectional study was conducted at the University of Gondar from August 15 to 25, 2021. A total of 422 study participants were selected using a stratified ~~simple~~ random sampling technique. A pretested self-administered questionnaire was employed to collect the data. Data were entered into EPI DATA 4.6 and exported to SPSS 25 for further analysis. Multivariable logistic regression analysis was performed to identify factors associated with student~~’~~s’ willingness to work in rural areas. The level of significance was decided based on the ~~AOR with a~~ 95 % confidence interval at a p-value of ≤ 0.05.

## Results

In this study, it was found that medical and health science student~~’~~s’ willingness to work in rural areas was 78.4% (95% CI: 74.3, 82.4). Being male (AOR = 2.15; 95% CI: 1.17, 3.94), having intention to continue with their profession (AOR = 2.5; 95% CI: 1.28, 4.86), having a favorable attitude towards working in rural areas (AOR = 7.32; 95% CI: 5.71, 18.65), and having a mother with no formal education (AOR = 2.23; 95% CI: 1.02, 4.85) and completed~~ing~~ primary education (AOR = 2.69; 95% CI: 1.1, 6.61) were significantly associated with willingness to work in rural areas.

## Conclusion

The willingness of students to work in rural areas was optimal. This calls for concerned bodies to create a conducive environment for male and female students to engage in rural areas without

hesitation. It is also important to ensure that students are willing to work in the rural areas voluntarily, instead of working in rural areas due to a lack of employment.

Keywords: Ethiopia, graduating students,~~willingness~~, ~~graduating students, ~~medical and health sciences, rural health services, willingness

# Introduction

The health workforce plays a key role in the provision of health. The equitable distribution of quality health personnel contributes significantly to ensur~~e~~ing the availability of healthcare services, irrespective of ~~their~~ location. Besides, it helps in progressing toward~~s~~ the Universal Health Coverage (UHC) goal by facilitating access to quality healthcare services for all (1).

More than 4 million health workers are needed to fill the gap in the global health workforce, as estimated by the  World Health Organization (WHO) (2). However, the geographical distribution of health workers is concentrated in affluent cities and regions worldwide, paying little attention to ~~take no notice of~~ the ~~–~~rural areas. Regardless of the level of economic development and organization of the health system, the disparity in the distribution of health workers ~~this model~~ exists in almost every country in the world, but this problem is particularly prominent in developing countries (3).

The shortage of health workers in rural areas has been identified as one of the biggest challenges for most countries to achieve the desired health-related goals. Developing countries suffer severely due to a significant shortage of health workers proportional to the population (4). Ethiopia is also one of the victims of this challenge, despite 84% of its citizen residing in ~~having about 84%~~ rural areas~~population~~ (5). One of the challenges to provide at least basic health services is the lack of trained health workers (6). In connection with~~to~~ this, the WHO recommends that at least 4.45 doctors, nurses, and midwives per 1000 population are needed to achieve the minimum levels of key health interventions. However, Africa has a 2.2 health workforce per 1000 population, which is often challenging to achieve the global goals (1).

In Ethiopia, the overall health professionals to population ratio were 1 per 1,000 population in 2018 (7), which is far below the minimum number estimated by the WHO. Hence, the healthcare system still suffers from the shortage mentioned earlier and the uneven distribution of healthcare workers between the urban and rural populations (8). The shortage of health staff also has a

significant impact on the healthcare delivery system such as the provision of essential life-saving interventions such as providing Expanded Program on Immunization (EPI), clean water, safe pregnancy, and childbirth services, and access to Human Immunodeficiency deficiency Virus/Acquired Immunodeficiency Syndrome (HIV/AIDS), tuberculosis (TB), and malaria treatment (9).

The Sustainable Development Goals (SDG) by United Nations (UN) member states in 2015, particularly goal 3 aim to ensure health and wellbeing for all. Target 3.1 specifically aims to reduce the Maternal Mortality Ratio (MMR) to less than 70 per 100,000, thereby saving the lives of one million mothers (10). In addition, 405,000 people died of malaria in 2018, 94% of which occurred in Africa (11). Moreover, an estimated 1.3 million TB-related deaths were recorded in 2017; additional 300,000 deaths among people living with HIV/AIDS (12). All these burdens are more common in rural areas. Shortage and mal-distribution of healthcare workers, particularly in rural areas will leading to failure to meet the SDGs. Hence, assigning adequate healthcare providers to these highly vulnerable groups will greatly help reduce the deaths of many lives.

Higher prevalence of mortality and morbidity are found in rural areas compared with urban areas (13). ~~Most of the morbidity and mortality are scattered in the rural areas.~~ Thus, the willingness of healthcare providers to work in rural areas is crucial to basically alleviating~~e~~ the problem. However, most health care providers are reluctant to work in rural settings. Some of the commonly cited reasons for not working in these areas are perceived low salary, unavailability of professional standards, unsatisfactory working environment, lack of access to skilled colleagues, and modern equipment (9). Besides, some others stated that learning and training opportunities are scarce when~~ile~~ they get employed in ~~the~~ rural areas (14). Furthermore, lack of the expected living conditions including electricity, water supply, and schools for their children, and having a plan to migrate out of their country after graduation are some of the reasons that lower the willingness to work in rural areas (14,15). Thus~~In this regard~~, ~~it is necessary to set problem-solving strategies by~~ it is important to give ~~providing~~ adequate remuneration to~~for~~ the~~ir~~ work for health workers, better professional training, and educational opportunities, and identify~~ing~~ effective and efficient human and non-human resources to meet the individual health needs of the underserved rural population.

Medical and health science student~~’~~s’ desire to go to the countryside will play an important role for the rural community that has been denied access to health services. Studies so far ~~Previous empirical evidence on student’s willingness to work in rural areas~~ revealed that students willingness to work in rural areas were 52% in China~~ 52%~~ (16), 45% in India~~ 45%~~ (17), 33.5% in Nigeria~~ 33.5%~~ (18), 55.4% in Ghana-~~55.4%~~ (19), and 29.7% in Ethiopia~~ 29.7%~~ (20). Being a male student (16,19), having a family member of lower academic attainments and lower socioeconomic status (21), rural background (22), and having a good attitude towards the rural community (15) had a positive impact on students willingness to work in the rural areas.

Although the health workforce in Ethiopia is growing, it is still less than half of the minimum number estimated by WHO. Increasing the willingness of graduating students by designing effective strategies will play a key role in reducing the burden of shortage of health care workers in the rural areas, thereby achieving the optimal health of the marginalized rural population. Besides, doing the research may offer basic evidence aimed at improving health workers’ needs and setting strategies considering the rural community. Therefore, this study ~~was~~ aimed to assess willingness to work in ~~the~~ rural areas and associated factors among graduating medicine and health science students at the University of Gondar, northwest Ethiopia.

# Methods and materials

## Study design, period, and study area

An institution-based cross-sectional study was conducted from August 1 to 25, 2021. The study was conducted at the University of Gondar, College of Medicine and Health Sciences, which is found in~~at~~ Gondar city, northwestern Ethiopia. The college was established in 1954 and is considered one of the oldest and most famous ~~u~~Universities in Ethiopia. There are 12 fields of study in health and medicine such as Nursing, Midwifery, Clinical laboratory, Pharmacy, Anesthesia, Psychiatry, Health informatics, Physiotherapy, Optometry, Environmental and occupational health, Public health officer (HO), and Medicine. Currently, there are 1068 graduating students from the 12 departments by~~on~~ September 2021.

## Study population

All graduating medical and health science students during the study period were the study population.

## Sample size determination and sampling procedure

The sample size for this study was calculated based on the assumptions of the single population proportion formula by considering the following assumptionsissues.: Since there is no similar study, we used the proportion of student's willingness to work in the rural area-50% (p = 0.5), level of significance- 5 % (α = 0.05), Z α/2 - 1.96, and margin of error - 5% (d = 0.05), and non - response rate -10. Therefore, the sample size was calculated as follows

$$n = \frac{(Z\alpha/2)^2 * p(1-p)}{d^2} = \quad n = \frac{(1.96)^{2*}0.5(1-0.5)}{(0.05)2} = 384.$$

After adding a 10% non-response rate, Tthe minimum adequate sample size was found to be 422., after adding a 10% non-response rate. There are 12 departments at the University of Gondar, college of medicine, and health sciences. All departments were included in the study. The lists of students were obtained from the registrar's office and the sampling frame was prepared by ordering the lists of students. Then, the total sample size was distributed proportionally to each department. Finally, the participants were selected using a stratified simple random sampling technique using a table of random generation **(Figure 1).**

## Variables of the study

The willingness of students to work in the rural areas was the outcome variable, whereas age, sex, place of origin, religion, professional category, paternal and maternal educational status, and paternal and maternal occupation, and personal behaviors like exposure to addictive substances, attitude to work in the rural area, and plan to emigrate were the independent variables.

## Measurements

**Willingness to work in rural areas:** Students were asked whether they have a willingness to work in rural areas or not after graduation. Students were asked whether they are willing to work in rural areas or not. A "yes" response who said "yes" when they are asked, whether they are

~~willing to work in the rural areas after graduation or not,~~ was considered ~~h~~as~~ a~~ willing~~ness~~ to work in the rural areas (19).

**Favorable attitude towards working in rural areas**: Students attitude towards working in rural areas was assessed using 11 questions: 1) Working in rural areas provide opportunities to use various skills 2) There is a supportive environment when working in a rural environment 3) Working in rural areas  limits communication with professional peers 4) Working in rural areas provide opportunities to work independently 5) There is lack of amenities and entertainment in rural areas 6) People in rural areas are friendly 7) Working in these areas causes isolation from family and friends 8) Working as a health care provider in hospitals or health centers in rural areas is the most important contribution to the health  of the population 9) Health science college prepared me well to work in rural areas 10) Working in rural hospitals areas is the most challenging 11) Working in rural hospitals provide opportunities for real life problem solving. Each question has five points Likert scale (1- strongly disagree, 2- disagree, 3- neutral, 4- agree, and 5- strongly agree). The minimum and maximum scores were 11 and 55, respectively. Thus, ~~Based on the summative score of variables designed to assess attitude-related questions, those~~ students who answered above the mean value are considered as having a favorable attitude (17).

## Data collection tools and procedures

The data collection tool was developed by reviewing the literature (16,17,19,20) and data were collected using a structured, pretested, and self-administered questionnaire. The questionnaire was evaluated by experts before data collection. The questionnaire contains socio-demographic characteristics and academic-related characteristics. Six BSc in midwifery graduating students and 1 MSc in Midwifery holder were recruited for data collection and supervision, respectively.~~, trained about the interview technique, collect and supervise the data collection process, respectively~~.

## Data quality control

To ensure consistency and understandability, the questionnaire was initially prepared in English and translated to the local Amharic language and back to English. A pretest was done on 5% of the calculated sample size at Bahir Dar University to check the response, language intelligibility, and relevance of the questionnaire. The data collectors and supervisors were trained for a day

regarding the overall data collection process. At the time of actual data collection, the collected data was checked daily by the supervisor for completeness.

## Data processing and analysis

Data were entered into EPI DATA 4.6 and exported to SPSS version 25 for further cleaning and analysis. Tables, figures, mean, and proportions are used to state the descriptive statistics of the study participants. The chi-square assumption was tested. Binary logistic regression was fitted to identify independent predictors. , and vVariables with a p-value of less than 0.25 in the bivariable logistic regression were included in the multivariable logistic regression to identify the final independent predictors. to control potential confounding factors. The chi-square assumption was tested and Hosmer-Lemeshow was used to test the model's goodness of fit. In addition, variance inflation factor (VIF), and standard error were used to screen multi-collinearity among independent variables. In the multivariable logistic regression analysis, a p-value of ≤ 0.05 and 95% CI for the adjusted odds ratio (AOR) were used to determine the significancet and degree of association between willingness to work in rural areas and the explanatory variables.

## Ethical considerations

The ethical clearance letter was obtained from the School of Midwifery under the delegation of the Institutional Review Board (IRB) of the University of Gondar. A formal administrative approval letter was gained from the college of medicine and health sciences. After clearly explaining the purpose of the study for to the students, anonymous written informed consent was obtained from each study participant. Interviewees have beenwere given the right to refuse and withdraw from the study at any time.

# Result

## Socio-demographic characteristics of study participants

A total of 422 study participants were recruited but only 402 of them completed the study, giving a response rate of 95.26%. The mean age (standard deviation) of the students was 23.9 years old (SD ± 2.28) and the majority (70.4%) of students were in the age group of < 25 years. Nearly

two-thirds (62.4%) of the study participants were males. About 29.4% of the participants were medical students and 46% of them were from urban areas by birthplace **(Table 1).**

## Academic, medical, and behavior related characteristics

~~From~~ Of the total study participants, nearly three-fourths (73.9%) believed that more job opportunities are available in ~~the~~ urban areas. About 58.5% of the study participants joined their first departmental choice and ~~have~~ got information regarding the department before campus entry. More than two-thirds (68.4%) and fourth-fifths (81.1%) of the study participants~~subjects~~ had a favorable attitude towards working in rural areas and intended to stay in their profession, respectively **(Table 2).**

## Willingness to work in rural areas and associated factors among graduating students

The willingness of students to work in rural areas was 78.4% (95% CI: 74.3, 82.4). In the bivariable logistic regression, sex, age, mother's educational status, father's educational status, place of birth, intention to continue with the profession, and attitude towards working in the rural areas were associated with willingness to work in rural areas. However, ~~Besides~~, in the multivariable logistic regression analysis ~~revealed that~~ being male, ~~had an~~ intention to continue with the profession, having~~d~~ a mother with no formal education and attended~~ing~~ primary education, and having a favorable attitude towards working in rural areas were variables ~~having a~~ significantly associated~~ion~~ with willingness to work in rural areas.

Hence, the odds of having a good willingness to work in rural areas ~~was~~ were two times higher among male students (AOR = 2.15; 95% CI: 1.17, 3.94) as compared to female students. Similarly, students who had an intention to continue with their profession were 2.5 times higher (AOR = 2.5; 95% CI: 1.28, 4.86) compared with those students who had ~~n't have~~no intention. Likewise, the likelihood of having a willingness to work in rural areas was 7.32 times higher (AOR = 7.32; 95% CI: 5.71, 18.65) among students who had a favorable attitude towards working in rural areas as compared to their counterparts. Lastly, students who had a mother with no formal education and attended~~ing~~ primary education were 2.23 (AOR = 2.23; 95% CI: 1.02, 4.85) and 2.69 times (AOR = 2.69; 95% CI: 1.1, 6.61) more likely to have had a willingness to

work in rural areas as compared to students who had a mother attended~~ing~~ college and above education, respectively (**Table 3**).

## Perceived reasons for not willing to work in rural areas

About 21.6% (87) of the study participants were not willing to work in rural areas after graduation. The frequently mentioned reasons by the students for not ~~have a willingness~~ to work in rural areas include lack of infrastructure in rural areas, not to be far from family members, and peace and security issues **(Figure 2).**

# Discussion

This study aimed to assess the willingness to work in rural areas, and associated factors among graduating medical and health science students at the University of Gondar, northwest Ethiopia. ~~Thus, more than three-fourths of students were willing to work in rural areas. In addition, being male, having an intention to continue with their profession, having a favorable attitude towards working in rural areas, and having a mother with no formal education and attending primary education were factors associated with student's willingness to work in rural areas~~.

This study found that student's willingness to work in rural areas was 78.4%, which was far higher than studies conducted in Egypt-52.3% (14), China-52% (16), India-45% (17), Nigeria-33.5% (18), Ghana-55.4% (19), and Ethiopia-29.7% (20). The possible explanation for the variation might be differences in socio-demographic contexts and time gaps. More than 80% of Ethiopia's population lives in rural areas (5), which may need more health workers, and new job opportunities in the health care system are available there. Besides, the number of health workers might be limited so far, ~~there may not be many health professionals before,~~ and jobs might be available ~~no one wants to go to the countryside to work because there was no shortage of jobs in~~ in the urban areas.~~, previously~~. Nowadays, however, there might~~ay~~ be a higher number of health professionals than ever before, and jobs may not be easy to find, especially in Ethiopia.~~, s~~So newly graduating students may want to work in rural areas unreservedly. In light of this, the Ethiopian government and other stakeholders should address and implement conditions for job creations for medical and health science students, and arrange opportunities that will favor students to work in rural areas in all aspects. In turn, this idea will reduce the student's intention to leave their profession and burnout in the workplace.

Regarding factors associated with willingness to work in rural areas, this study affirmed that the sex of students affects their willingness to work in rural areas. Accordingly, male students were two times more likely to have had a good willingness to work in rural areas as compared to their female counterparts. This finding is consistent with studies conducted in Egypt and Ghana (16,19). This can be explained by the fact that male students might cope with problems that are considered to be present in rural areas, such as transportation problems, and lack of electricity, and lack of safe water.~~, etc.~~ ~~This is because w~~When there is a lack of transportation~~ and a scarcity of daily consumption~~, health workers ~~there~~ may ~~be a need to~~ walk to the city on foot.~~,~~ Thus, ~~and~~ females may be afraid that they will be in trouble. However, this finding contradicts a study conducted in China that founds female students are ~~more~~ intend to work more in rural areas compared with their male counterparts (21). This could be justified by differences in the country's infrastructure and culture of the community. In Ethiopia, the infrastructure might not be ~~propitious~~favorable for females like that of China. Besides, female students might not be willing to be far from families because of the perception of the community, in which the community may not believe ~~ing~~ females ~~might not being able to~~can deal with the difficult environment. ~~they might face~~.

This study revealed that students who had a mother with no formal education and had attended ~~ing~~ primary education were 2.23 and 2.69 times more likely to have had a willingness to work in rural areas as compared to students who had a mother attended~~ing~~ college and above education, respectively. This finding is in agreement with a study conducted in China (21). This could be explained by parents might have a big influence on shaping and directing the attitude ~~and behaviors~~and behaviors of their children, including choosing departments and workplaces.~~educations what to learn and work something else~~. Besides, those students who had a family with less educational achievement are expected to be less affluent. Thus, to support the household economy, students will have a willingness to work in rural areas irrespective of preconditions.

In this study, the odds of willingness to work in rural areas were ~~as~~ two times higher among students who had plans to continue with their profession as compared to those students who had no ~~n't~~ intention ~~ded~~ to continue with the profession that they have currently. A study conducted in Nigeria reported a similar result (23). This could be explained by the fact that students who

intend to continue with their profession may initially join the department by their choice and could have a strong commitment to work in any part of the country. On the other hand, students may not have the interest to continue with their current profession due to different reasons like a plan to immigrate to foreign counties and engage in other business. Because those students with no intention to continue with their profession might have a plan to participate in other income-generating activities and plan to change their profession due to a lack of interest in the department after they are engaged~~ in~~. In this regard, the Ethiopian government should pay attention to the healthcare providers by settling different encouraging undertakings such as crafting job opportunities, evenhanded salary for their work, and timely educational opportunities~~chances~~ for their vertical growth.

This study also ~~was~~ found that students' attitude~~s~~ toward~~s~~ rural work affected their willingness to work in rural areas after graduation. Accordingly, students with a positive attitude towards working in rural areas were seven times more likely than students with unfavorable attitudes. This result is consistent with a study conducted in Ethiopia (15). The attitude of an individual has~~ve~~ a direct impact on~~in~~ determining responses to situations, psychological preparations, and influencing responses to all situations around them (17). Thus, students who have a positive attitude to the need for rural community support and access to equitable health care may want to work in rural areas unconditionally. They might also be happy to help people, and ~~they may be~~ able to fulfill their good wishes by serving the rural people, where highly in need of healthcare access. Lastly, we are pleased to acknowledge some of the limitations of this study. Due to the cross-sectional nature of the study design, it may not be possible to know the exact effect of the identified factors on the outcome variable. Moreover, since the questionnaire was self-administered, response bias might be introduced. However, before data collection, students were told that their participation in the research is significant and that they would fill in the required information honestly for the success of the research.

# Conclusion

More than three-quarters of medical and health science students were willing to work in rural areas. Being a male student, having the intention to stay in their profession, having a good attitude towards working in rural areas,~~ having a good attitude towards working in rural areas~~,

and students having a mother of low academic achievement ~~was~~ were observed to affect the student's willingness to work in the rural areas. Thus, this calls for health policymakers and other stakeholders to give attention to arranginge all-encompassing job opportunities in the rural areas that will favor both males and females. In addition, it is important to build a good view and understanding for students about rural areas during their campus life. ~~Moreover, benefits improvement is essential for students to stay in their profession without getting disappointed and planned to leave their profession.~~

## Supporting information

S1 Questionnaire: English and Amharic version.

S2 Dataset: SPSS data.

# Authors' Contributions

**Conceptualization**: Azmeraw Ambachew Kebede

**Data curation**: Blen Getachew, Biruk Bizuneh, Birhanu Ewunetu, Dawit Kassahun, Dessalegn Fentahun, Destaw Ademe, Endeshaw Admasu Cherkos, Azmeraw Ambachew Kebede

**Formal analysis:** Blen Getachew, Biruk Bizuneh, Birhanu Ewunetu, Dawit Kassahun, Dessalegn Fentahun, Destaw Ademe, Endeshaw Admasu Cherkos, Azmeraw Ambachew Kebede

**Investigation:** Blen Getachew, Biruk Bizuneh, Birhanu Ewunetu, Dawit Kassahun, Dessalegn Fentahun, Destaw Ademe, Endeshaw Admasu Cherkos, Azmeraw Ambachew Kebede

**Methodology:** Blen Getachew, Biruk Bizuneh, Birhanu Ewunetu, Dawit Kassahun, Dessalegn Fentahun, Destaw Ademe, Endeshaw Admasu Cherkos, Azmeraw Ambachew Kebede

**Validation:** Blen Getachew, Biruk Bizuneh, Birhanu Ewunetu, Dawit Kassahun, Dessalegn Fentahun, Destaw Ademe, Endeshaw Admasu Cherkos, Azmeraw Ambachew Kebede

**Visualization:** Blen Getachew, Biruk Bizuneh, Birhanu Ewunetu, Dawit Kassahun, Dessalegn Fentahun, Destaw Ademe, Endeshaw Admasu Cherkos, Azmeraw Ambachew Kebede

**Writing-original draft:** Blen Getachew and Azmeraw Ambachew Kebede

**Writing- review and editing:** Blen Getachew, Biruk Bizuneh, Birhanu Ewunetu, Dawit Kassahun, Dessalegn Fentahun, Destaw Ademe, Endeshaw Admasu Cherkos, Azmeraw Ambachew Kebede


# Acknowledgments

We would like to thank the University of Gondar for providing study ethical clearance to conduct this study. Our gratitude also goes to all data collectors and study participants. We are glad to Hospitals in Gondar province for writing permission letter.


**Data Availability Statement**

All relevant data are within the manuscript and its Supporting information files.


**Funding:** The author(s) received no specific funds for this work.

**Competing interests**: The authors declare that they have no conflict of interest in this work


**Abbreviations**

AOR; Adjusted Odds Ratio, CI; Confidence Interval, COR Crude Odds Ratio, SPSS; Statistical Package for Social Science, SSA; Sub-Saharan Africa, WHO; World Health Organization

Table 1: Socio-demographic characteristics of study participants in the University of Gondar, college of medicine and health sciences, northwest Ethiopia, 2021 (n = 402)

| Charactestics | Frequency | Percentage (%) |
|---|---|---|
| **Age in year** | | |
| ≥≤25 | 283 | 70.4 |
| ≥25 | 119 | 29.4 |
| **Sex** | 251 | 62.4 |
| Male | 151 | 37.6 |
| Female | | |

| | | |
|---|---|---|
| **Place of birth** | | |
| Urban | 185 | 46 |
| Semi-urban | 59 | 14.7 |
| Rural | 158 | 39.3 |
| **Religion** | | |
| Orthodox Christian | 344 | 85.6 |
| Protestant | 42 | 10.4 |
| Others[a] | 16 | 4 |
| **Current marital status** | | |
| Married | 19 | 4.7 |
| In relationship | 31 | 7.7 |
| Single | 352 | 87.6 |
| **Average monthly pocket money** | | |
| < 500 ETB | 70 | 17.4 |
| 500-999 ETB | 108 | 26.7 |
| ≥ 1000 ETB | 224 | 55.7 |
| **Department** | | |
| Medicine | 118 | 29.4 |
| Nursing | 43 | 10.7 |
| Midwifery | 33 | 8.2 |
| Public health officer | 32 | 8 |
| Psychiatry | 17 | 4.2 |
| Environmental and occupational health | 21 | 5.2 |

| | | |
|---|---|---|
| Optometry | 17 | 4.2 |
| Physiotherapy | 17 | 4.2 |
| Health informatics | 20 | 5 |
| Anesthesia | 18 | 4.5 |
| Pharmacy | 35 | 8.7 |
| Medical laboratory | 31 | 7.7 |
| **Average monthly income of the family** | | |
| <1500 ETB | 14 | 3.5 |
| 1501-5000 ETB | 115 | 28.6 |
| ≥ 5001ETB | 273 | 67.9 |
| **Mothers educational status** | | |
| No formal education | 159 | 39.6 |
| Primary education | 83 | 20.6 |
| Secondary education | 67 | 16.7 |
| Diploma and above | 93 | 23.1 |
| **Mother's occupation** | | |
| Housewife and/or farmer | 229 | 57 |
| Government employee | 67 | 16.7 |
| Self employed | 47 | 11.7 |
| Merchant | 57 | 14.2 |
| Others[b] | 2 | 0.5 |

| | | |
|---|---|---|
| **Father's educational status** | | |
| No formal education | 110 | 27.4 |
| Primary | 120 | 29.9 |
| Secondary | 47 | 11.7 |
| Diploma and above | 125 | 31.1 |
| **Fathers occupation** | | |
| Farmer | 168 | 41.8 |
| Merchant | 74 | 18.4 |
| Government employed | 105 | 26.1 |
| Self employed | 53 | 13.2 |
| Others[b] | 2 | 0.5 |

a = Muslim and catholic, b = retired, ETB- Ethiopian Birr

Table 2: Academic, medical, and behavioral related characteristics of study participants in the University of Gondar, college of medicine and health sciences, northwest Ethiopia, 2021 (n = 402)

| Charactestics | Frequency | Percentage (%) |
|---|---|---|
| **History of ~~c~~khat chewing** | | |
| Yes | 33 | 91.8 |
| No | 369 | 8.2 |
| **History of cigarette smoking** | | |
| Yes | 23 | 5.7 |
| No | 379 | 94.3 |
| **Ever drunk alcohol** | | |
| Yes | 178 | 55.7 |

| | | |
|---|---|---|
| No | 224 | 44.3 |
| **Getting information about the department before campus entry** | | |
| Yes | 240 | 59.7 |
| No | 162 | 40.3 |
| **Source of information (n = 240)** | | |
| Friends | 116 | 48.3 |
| Healthcare providers | 17 | 7.1 |
| Families | 61 | 25.4 |
| Mass media | 46 | 19.2 |
| **Is the department your first choice** | | |
| Yes | 235 | 58.5 |
| No | 167 | 41.5 |
| **Intention to continue with the profession** | | |
| Yes | 329 | 81.1 |
| No | 73 | 18.2 |
| **Attitude towards working in rural areas** | | |
| Favorable | 275 | 68.4 |
| Unfavorable | 126 | 31.1 |
| **Where did you think jobs are available** | | |
| Urban | 297 | 73.9 |
| Rural | 105 | 26.1 |
| **Do you have any known medical disorder** | | |

| | | |
|---|---|---|
| Yes | 384 | 95.5 |
| No | 18 | 4.5 |

Table 3: Bivariable and multivariable logistic regression analysis of factors associated with graduating students willingness to work in rural areas in the University of Gondar, northwest Ethiopia, 2021 (n = 402)

| Variables | Willingness to work in rural areas | | COR ( 95% CI) | AOR ( 95% CI) |
|---|---|---|---|---|
| | Yes | No | | |
| **Sex** | | | | |
| Male | 214 | 37 | 2.86 (1.76, 4.65) | **2.15 (1.17, 3.94)*** |
| Female | 101 | 50 | 1 | 1 |
| **Age in year** | | | | |
| < 25 | 213 | 70 | 1 | 1 |
| ≥ 25 | 102 | 17 | 1.97 (1.10, 3.52) | 1.35 (0.66, 2.75) |
| **Place of birth** | | | | |
| Urban | 132 | 53 | 1 | 1 |
| Semi-urban | 46 | 13 | 1.42 (0.71, 2.84) | 0.92 (0.36, 2.31) |
| Rural | 137 | 21 | 2.62 (1.49, 4.58) | 1.61 (0.63, 4.12) |
| **Intention to continue with the profession** | | | | |
| Yes | 267 | 62 | 2.24 (1.28, 3.91) | **2.50 (1.28, 4.86)*** |
| No | 48 | 25 | 1 | 1 |
| **Mother's educational status** | | | | |
| No formal education | 137 | 22 | 2.54 (1.35,4.81) | **2.23 (1.02, 4.85)*** |
| Primary education | 72 | 11 | 2.68 (1.23, 5.82) | **2.69 (1.1, 6.61)*** |

| | | | | |
|---|---|---|---|---|
| Secondary education | 40 | 27 | 0.6 (0.31, 1.17) | 0.58 (0.268, 1.27) |
| Diploma and above | 66 | 27 | 1 | 1 |
| **Father's educational status** | | | | |
| No formal education | 96 | 14 | 3.35 (1.70, 6.56) | 3.35 (1.00, 10.36) |
| Primary education | 102 | 18 | 2.76 (1.48, 5.16) | 1.62 (0.66, 4.01) |
| Secondary education | 33 | 14 | 1.15 (0.55, 2.38) | 1.03 (0.39, 2.72) |
| Diploma and above | 84 | 41 | 1 | 1 |
| **Attitude towards working in the rural area** | | | | |
| Favorable | 249 | 26 | 8.7 (5.10, 14.85) | **7.32 (5.71,18.65)**** |
| Unfavorable | 66 | 60 | | 1 |

Notes: * $P \leq 0.05$, ** $P \leq 0.001$

Abbreviations: AOR, adjusted odds ratio; COR, crude odds ratio; CI, confidence interval; 1, reference category

**Reference**


1. World Health Organization. (2016). Health workforce requirements for universal health coverage and the Sustainable Development Goals. (Human Resources for Health Observer, 17). World Health Organization. https://apps.who.int/iris/handle/10665/250330.

2. World Health Organization. (2006). The world health report: 2006: Working Together for Health. World Health Organization. Available from: https://apps.whoint/iris.handle/10665/43432

3. Asante AD, Zwi AB. Factors influencing resource allocation decisions and equity in the health system of Ghana. 2009;123(5):371-377. DOI: https://doi.org/10.1016/j.puhe.2009.

4. Liu JX, Goryakin Y, Maeda A, Bruckner T, Scheffler R. Global Health Workforce Labor Market Projections for 2030. Hum Resour Health. 2017;15(1. DOI: 10.1186/s12960-017-0187–2).

5. Assefa Y, Hill PS, Gilks CF, Admassu M, Tesfaye D, Van Damme W. Primary health care contributions to universal health coverage, Ethiopia. Bull World Health Organ. 2020;98(12.):894-905.DOI: 10.2471/BLT.19.248328.

6. Mengistu BS, Vins H, Kelly CM, McGee DR, Spicer JO, Derbew M, et al. Student and faculty perceptions on the rapid scale-up of medical students in Ethiopia. BMC Med Educ. 2017;17(1):1-8. DOI:10.1186/s12909-016-0849–0.

7. Health Sector Transformation Plan II 2020/2021-2024/2025. Ethiop Minist Heal [Internet]. 2021;25:1–128. Available from: e-library.moh.gov.et/library/index/php/knoledge-product/health -sector-transformation-plan-ii-sstp-ii-2020-21-2024-25/

8. Bradley E, Thompson JW, Byam P, Webster TR, Zerihun A, Alpern R, et al. Access and quality of rural healthcare: Ethiopian Millennium Rural Initiative. Int J Qual Heal Care. 2011;23(3):222-230. DOI:10.1093/intqhc/mzr013.

9. Girasek E, Eke E, Szócska M. Analysis of a survey on young doctors’ willingness to work in rural Hungary. Hum Resour Health. 2010;8(1):1-6. DOI: 10.1186/1478-4491-8–13.

10. SDG. Final list of proposed Sustainable Development Goal indicators. Report of the Inter-

Agency and Expert Group on Sustainable Development Goal Indicators (E/CN.3/2016/2/Rev.1). 2016; Available from: https://sustainabledevelopment.un.org/content/documents/11803Official-List-of-Proposed-SDG-Indicators.pdf

11. Regional and global trends in burden of malaria cases and deaths. World Health Organization malaria report 2019. Available from: https://www.who.int/news-room/feature-stories/detail/world-malaria-report-2019.

12. Global tuberculosis report 2018. Geneva: World Health Organization; 2018. Licence: CC BY-NC-SA 3.0 IGO.

13. Curtin SC, Spenncer MR. Trends in death rates in urban and rural areas: United States, 1990-2019. NCHS Data Brief, no 417. Hyattsville, MD: National Center for Health Statistics. 2021. DOI: https://dx.doi.10.15620/cdc.109049.

14. Mohamed AM. Willingness and professional motivations of medical students to work in rural areas: a study in Alexandria, Egypt. Healthc Low-resource Settings. 2013;1(1):DOI: 10.4081/HLS.2013.E4.

15. Deressa W, Azazh A. Attitudes of undergraduate medical students of Addis Ababa University towards medical practice and migration, Ethiopia. BMC Med Educ. 2012;12(1):DOI: 10.1186/1472-6920-12-68.

16. Liu J, Zhu B, Mao Y. Association between rural clinical clerkship and medical students' intentions to choose rural medical work after graduation: A cross-sectional study in western China. PLoS One. 2018;13(4):DOI: 10.1371/JOURNAL.PONE.0195266.

17. Rajesh Kumar Singh, CMS Rawat SP. Attitude of medical students toward serving in rural areas and its determinants: a cross-sectional study from Uttarakhand. Int J Med Sci Public Heal. 2015;4(6):8-14.DOI: 10.5455/ijmsph.2015.03032015166.

18. Ndu AC, Aniwada EC, Umeh EN, Uzo NM, Uzoma CE. Perception and willingness of medical students towards working in rural communities. Niger J Med. 2019;28(3):275-280. DOI:10.4314/njm.v28i3.

19. Agyei-Baffour P, Kotha SR, Johnson JC, Gyakobo M, Asabir K, Kwansah J, et al.

Willingness to work in rural areas and the role of intrinsic versus extrinsic professional motivations - A survey of medical students in Ghana. BMC Med Educ. 2011;11(1):1-8. DOI: 10.1186/1472-6920-11–56.

20. Assefa T, Haile Mariam D, Mekonnen W, Derbew M. Medical students' career choices, preference for placement, and attitudes towards the role of medical instruction in Ethiopia. BMC Med Educ. 2017;17(1):1-10. DOI: 10.1186/s12909-017-0934-z.

21. Liu J, Zhang K, Mao Y. Attitude towards working in rural areas: a cross-sectional survey of rural-oriented tuition-waived medical students in Shaanxi, China. BMC Med Educ 2018 181. 2018;18(1):1-11. DOI: 10.1186/S12909-018-1209-Z.

22. Serneels P, Montalvo JG, Pettersson G, Lievens T, Buterae JD, Kidanu A. Who wants to work in a rural health post? The role of intrinsic motivation, rural background and faith-based institutions in Ethiopia and Rwanda. Bull World Health Organ. 2010;88(5):342-349. DOI: 10.2471/BLT.09.072728.

23. E N Ossanai, A F Una, R C Onyenakazi EUN. Emigration Plans after Graduation of Clinical Medical Students of Ebonyi State University Abakaliki, Nigeria: Implications for Policy. Niger J Clin Pract. 2020;23(7):995-1003. DOI: 10.4103/njcp.njcp.

**July 7, 2022**

**Manuscript ID:** PONE-D-21-30311 **(**Willingness to work in rural areas and associated factors among graduating medical and health science students at the University of Gondar, northwest Ethiopia, 2021)

**Response to Reviewers**

First of all, we would like to thank the reviewers and the editor for their constructive comments and valuable improvements to the manuscript. We have incorporated all the suggestions and comments. The changes made during revision have been shown with tracked changes and a point-by-point response-please see below.

**Response to the Editor(s) and journal office**

| S.N | Comments | Responses |
|---|---|---|
| 01 | Please include additional information regarding the survey or questionnaire used in the study and ensure that you have provided sufficient details that others could replicate the analyses. For instance, if you developed a questionnaire as part of this study and it is not under a copyright more restrictive than CC-BY, please include a copy, in both the original language and English, as Supporting Information. If the original language is written in non-Latin characters, for example Amharic, Chinese, or Korean, please use a file format that ensures these characters are visible. | Thank you, dear editor. The questionnaire has been developed after reviewing literature. The questionnaire has also been evaluated by experts before data collection. Bothe English and Amharic version of the questionnaire has been uploaded during revision. |
| 02 | In your Data Availability statement, you have not specified where the minimal data set underlying the results described in your manuscript can be found | Thank you, dear reviewer. All relevant data are within the manuscript and its Supporting information files (SPSS and questionnaire). |
| 03 | Please upload a copy of Figure 2, to which you refer in your text on page 16. If the figure is no longer to be included as part of the submission please remove all reference to it within the text | Thank you, dear reviewer. The two figures were in one word file. Now, we have uploaded the two figures separately. |

**Response to Reviewer(s) comments**

**Reviewer #1**

| S.N | Comments | Responses |
|---|---|---|
| 01 | I believe the use of the heterogeneous group of different cadres of health personnel is not adequate for the following reasons:<br>1.1 WHO in its world health report 2006 (World Health Organization. (2006). The world health report: 2006: working together for health) has clearly defined health professionals to include doctors, nurses and midwife in training hence comparison cannot be made lumping up other classes of health workers in training who ordinarily may not be heavily impacted by the issue under consideration given that their services are not always primarily needed as core areas for health provision mandate in rural areas. | Thank you, dear reviewer, for your deep review and thoughtful comment. You are right, according to the WHO 2006 report, the frontline healthcare providers for the rural community are nurses, midwives, and medical doctors. However, it is not entirely mean that the other professionals/professions are needed in rural areas. It is well known that majority of the health workers in rural areas are the abovementioned professions. Though the number matters, every profession is needed in rural areas as far as health facilities and the problem is there. For example, a primary hospital may need 20 nurses, 10 midwives and 1o medical doctors. It may also need 2 optometry, 2 physiotherapy, 2 psychiatry, 1 health informatics, etc, professionals. Thank you for the understanding |
| 02 | The previous works cited and compared with your results clearly have defined their works with a specific group of class of health worker student's usually medical students.<br><br>Given the difference in training, scope, primary job deployment (e.g. PHC workers for rural areas) and their need in rural areas amongst others, it may be difficult to lump all the classes of health workers student together. Thus given the foregoing it may be necessary to substantially redo the recruitment and focus either on health professional's student - medical and nursing | Thank you, dear reviewer. Yes you are right, previously studies use medical students only. As we try to describe above, all professions are need though the number matters. |

| | | |
|---|---|---|
| | students or the other cadres who ordinarily are expected to be found in the rural areas in the PHC. Then the analysis will focus on either of the group with a new calculated sample size. | |
| 03 | Can you be more explicit and outline the attitude related questions making reference to the questionnaire in the method section? Again, a write up including some diagram or table for illustration demonstrating the derivation of the attitude summation scores from the question and its final categorization would be in order as already provided from the questionnaire. | Thank you dear reviewer. We did based on your kind recommendation. Please kindly see the revised manuscript. |
| 04 | Why the use of 0.2 for test of significance for the bivariable, any reference or rationale for the choice? | Thank you, dear reviewer, for your thoughtful concern. Please accept our apologies, it was typing error. The basis was to take all variables having a p-value of <0.25 to set for the multivariable regression. However, it was written < 0.2 in the document and it was typing error. We did based on the assumption to include all variables having a p-value of <0.25 as evidenced by existing literature (http://dx.doi.org/10.21037/atm.2016.02.15, doi: 10.1186/1751-0473-3-17, doi: 10.1186/1751-0473-3-17). For this study, we used a p-value of 0.25 assumption to proceed to the multivariable logistic regression. It has been corrected in the revised manuscript. Thank you once again for raising this important question. |
| 05 | What is the difference between bivariable logistic regression and chi-square and the multivariable logistic regression? How the final model was for the multivariable created i.e. which variables were selected and added into that final model? Did all the variables in the bivariable | A chi-square statistic is a measure of the difference between the observed and expected frequencies of the outcomes of a set of events or variables. Chi-square can be used to test whether two variables are related or |

| | | |
|---|---|---|
| | meet the p-value cutoff you stated you used in moving to the multivariable regression? | independent from one another. It can also be used to test the goodness-of-fit between an observed distribution and a theoretical distribution of frequencies<br><br>The bivariable logistic regression is a model used to test whether a single independent variable and the outcome variable has an association. Whereas, the multivariable logistic regression is a model used to identify the significantly associated variables after introducing many independent variables at a time and a single independent variable. In this study, the variables put in the regression table meet the p-value cutoff point. |
| 06 | What was only displayed in the table was the COR and AOR with p-value only for the AOR. I think it may be good to make this much clearer. | Thank you, dear reviewer. Since the CI is more descriptive, we only indicate p-values for significantly associated variables. |
| 07 | It would have been nice to add some write up on the results from the bivariable analysis before detailing on the multivariable adjusted ones | Thank you, dear reviewer. We did it in the revised manuscript. Please kindly see the revised manuscript. |
| 08 | There is a need to rework and format your reference list for consistency as some had missing author's name (e.g. ref. 23), more authors than the original paper (e.g. ref3), wrong citation (ref 2), typos, etc. It would also be nice to add the doi for those articles that have | Thank you, dear reviewer. We went through the entire reference and revised it based on your kind suggestion. Besides, the DOI has been added for those articles that have. Please kindly take a look at the revised manuscript. |
| 09 | Please can you explain the choice of the use of 50% for previous prevalence (p) for the sample size calculation? Can you provide the reference or provide justification for the choice | Thank you, dear reviewer. Since there was no recent similar study, we used 50%. A sentence describing this has been added in the revised manuscript. |
| 10 | There are lots of grammatical errors that may need the article to be copy edited to improve on its language | Thank you, dear reviewer, for your kind suggestions and valuable help. We went through the whole document and look for |

| | | |
|---|---|---|
| | structure. I have highlighted a few in the body of the article text. | grammar errors. The highlights you made were beyond crucial. Thank you once again. |
| 11 | Is it stratified simple random sampling or stratified random sampling? | Thank you, dear reviewer. It is stratified random sampling and corrected in the revised manuscript. |
| 12 | Other minor issues and comments especially for grammatical issues are as contained in my comments and highlight in the article | Thank you, dear reviewer. Corrected in the revised manuscript. |
| 13 | Suggestions and comments in the body of the article text | All the suggestions and comments on the PDF are clearly addressed. Please kindly see the revised manuscript. Thanks once again for your priceless contribution to the improvements of this manuscript. |

**Reviewer #2**

| S.N | Comments | Responses |
|---|---|---|
| 01 | Very interesting article. This is a relevant social and medical issue that must be discussed | Thank you, dear reviewer, for your support and valuable contribution to the improvement of the manuscript. |
| 02 | The keywords do not reflect the purpose of the article - for example, the MeSH term "Rural Health Services" could be included | Thank you, dear reviewer. It has been included in the revised manuscript. |
| 03 | It's necessary to revise the text. For example: - "The willingness of students to work in the rural areas was the outcome variable, while age, sex..." - Incomplete sentence | Thank you, dear reviewer. It has been corrected in the revised manuscript. |
| 04 | Students were asked whether they have a willingness to work in rural areas or not after graduation. Students who said “yes” when they are asked, whether they are willing to work in the rural areas after graduation or not, was considered..." – Repetition | Thank you, dear reviewer. It has been revised, please see the revised manuscript. |

| | | |
|---|---|---|
| 05 | who hadn’t have intention..." - Wrong past tense who hadn’t have intention..." - Wrong past tense | Thank you, dear reviewer. We did a revision, please see the revised manuscript. |
| 06 | Besides, the multivariable logistic regression analysis revealed that being male, had an intention to continue with the profession, had a mother with no formal education..." - Wrong tense | Thank you, dear reviewer. A revision has been made. |
| 07 | were variables having a significant association" - Wrong tense | Thank you, dear reviewer. We did a correction in the revised manuscript. |
| 08 | This could be explained by parents might have a big influence on shaping and directing the attitude and behaviors of their children, including educations what to learn and work something else." - Educations what to learn? | Thank you, dear reviewer. We did a revision, please see the revised manuscript |
| 09 | "as compared to those students who hadn’t intended to continue with the profession that they have currently- Wrong past tense | Thank you, dear reviewer. We did a revision based on your kind suggestion |
| 10 | Because those students with no intention to continue with their profession might have a plan to participate..." - incomplete sentence | Thank you, dear reviewer. “Because those students with no intention to continue with their profession might have a plan to participate in other income-generating activities and plan to change their profession due to lack of interest in the department after they engaged in”. The sentence is what we write above. |
| 11 | This study also was found that students’ attitudes" - Also was found?<br>- "having a mother of low academic achievement was observed to affect" --> were observed | Thank you, dear reviewer. It has been checked and revised based on your kind recommendation. |
| 12 | The first paragraph of the discussion was the repetition of the results. | Thank you, dear reviewer. We did a revision and the text has been removed due to the repetition. |

| | | |
|---|---|---|
| 13 | In the conclusion, "having a good attitude towards working in rural areas" was repeated. Also "Moreover, benefits improvement is essential for students to stay in their profession without getting disappointed and planned to leave their profession." was not raised in the discussion. | Thank you, dear reviewer. The repetition has been deleted and the last sentence has also been deleted. |
| 14 | Table 1: > 25 ≥25 --> I think it was < 25 and ≥25 | Thank you, dear reviewer. It was typing error and corrected in the revised manuscript. |
| 15 | The monthly income (Average monthly pocket money) is in what currency? Dollar? | Thank you, dear reviewer. Please accept our apologies for problem caused, it was typing error. The currency is in Ethiopian Birr (ETB). |
| 16 | Table 2: "chat chewing" --> isn't khat chewing? | Thank you, dear reviewer. It has been corrected. |
| 17 | You don't need to put rows for "yes" and "no" in the table. You can just put "yes". Example:<br>History of chat chewing, Yes 33 91.8 | Thank you, dear reviewer. Dear reviewer, you are right it is possible to proceed as per your kind recommendation. If you don't mind, we prefer to continue as it is to make it clear the reader and no confusion will create to the audience. |
| 18 | English must be revised. However, I reinforce the value of the article and thematic | Thank you, dear reviewer, for your priceless contribution you made for the improvements of the manuscript. We went through the whole document for English language and grammars. Please kindly see the revised manuscript. |
