# Supplementary material for: Willingness to work in rural areas and associated factors among graduating health students at the University of Gondar, northwest Ethiopia, 2021
Source: PLoS One. 2022 Oct 21;17(10):e0276594. doi: 10.1371/journal.pone.0276594 (PMC9586395; doi:10.1371/journal.pone.0276594)
Supplement: Attachment — Submitted filename: Response to Reviewers.docx [file pone.0276594.s004.docx]

**July 7, 2022**

**Manuscript ID:** PONE-D-21-30311 **(**Willingness to work in rural areas and associated factors among graduating medical and health science students at the University of Gondar, northwest Ethiopia, 2021)

**Response to Reviewers**

First of all, we would like to thank the reviewers and the editor for their constructive comments and valuable improvements to the manuscript. We have incorporated all the suggestions and comments. The changes made during revision have been shown with tracked changes and a point-by-point response-please see below.

**Response to the Editor(s) and journal office**

| S.N | Comments | Responses |
| --- | --- | --- |
| 01 | Please include additional information regarding the survey or questionnaire used in the study and ensure that you have provided sufficient details that others could replicate the analyses. For instance, if you developed a questionnaire as part of this study and it is not under a copyright more restrictive than CC-BY, please include a copy, in both the original language and English, as Supporting Information. If the original language is written in non-Latin characters, for example Amharic, Chinese, or Korean, please use a file format that ensures these characters are visible. | Thank you, dear editor. The questionnaire has been developed after reviewing literature. The questionnaire has also been evaluated by experts before data collection. Bothe English and Amharic version of the questionnaire has been uploaded during revision. |
| 02 | In your Data Availability statement, you have not specified where the minimal data set underlying the results described in your manuscript can be found | Thank you, dear reviewer. All relevant data are within the manuscript and its Supporting information files (SPSS and questionnaire). |
| 03 | Please upload a copy of Figure 2, to which you refer in your text on page 16. If the figure is no longer to be included as part of the submission please remove all reference to it within the text | Thank you, dear reviewer. The two figures were in one word file. Now, we have uploaded the two figures separately. |

**Response to Reviewer(s) comments**

**Reviewer #1**

| S.N | Comments | Responses |
| --- | --- | --- |
| 01 | I believe the use of the heterogeneous group of different cadres of health personnel is not adequate for the following reasons: 1.1 WHO in its world health report 2006 (World Health Organization. (‎2006)‎. The world health report: 2006: working together for health) has clearly defined health professionals to include doctors, nurses and midwife in training hence comparison cannot be made lumping up other classes of health workers in training who ordinarily may not be heavily impacted by the issue under consideration given that their services are not always primarily needed as core areas for health provision mandate in rural areas. | Thank you, dear reviewer, for your deep review and thoughtful comment. You are right, according to the WHO 2006 report, the frontline healthcare providers for the rural community are nurses, midwives, and medical doctors. However, it is not entirely mean that the other professionals/professions are needed in rural areas. It is well known that majority of the health workers in rural areas are the abovementioned professions. Though the number matters, every profession is needed in rural areas as far as health facilities and the problem is there. For example, a primary hospital may need 20 nurses, 10 midwives and 1o medical doctors. It may also need 2 optometry, 2 physiotherapy, 2 psychiatry, 1 health informatics, etc, professionals. Thank you for the understanding |
| 02 | The previous works cited and compared with your results clearly have defined their works with a specific group of class of health worker student’s usually medical students.  Given the difference in training, scope, primary job deployment (e.g. PHC workers for rural areas) and their need in rural areas amongst others, it may be difficult to lump all the classes of health workers student together. Thus given the foregoing it may be necessary to substantially redo the recruitment and focus either on health professional’s student - medical and nursing students or the other cadres who ordinarily are expected to be found in the rural areas in the PHC. Then the analysis will focus on either of the group with a new calculated sample size. | Thank you, dear reviewer. Yes you are right, previously studies use medical students only. As we try to describe above, all professions are need though the number matters. |
| 03 | Can you be more explicit and outline the attitude related questions making reference to the questionnaire in the method section? Again, a write up including some diagram or table for illustration demonstrating the derivation of the attitude summation scores from the question and its final categorization would be in order as already provided from the questionnaire. | Thank you dear reviewer. We did based on your kind recommendation. Please kindly see the revised manuscript. |
| 04 | Why the use of 0.2 for test of significance for the bivariable, any reference or rationale for the choice? | Thank you, dear reviewer, for your thoughtful concern. Please accept our apologies, it was typing error. The basis was to take all variables having a p-value of <0.25 to set for the multivariable regression. However, it was written < 0.2 in the document and it was typing error. We did based on the assumption to include all variables having a p-value of <0.25 as evidenced by existing literature (<http://dx.doi.org/10.21037/atm.2016.02.15>, doi: 10.1186/1751-0473-3-17, doi: 10.1186/1751-0473-3-17). For this study, we used a p-value of 0.25 assumption to proceed to the multivariable logistic regression. It has been corrected in the revised manuscript. Thank you once again for raising this important question. |
| 05 | What is the difference between bivariable logistic regression and chi-square and the multivariable logistic regression? How the final model was for the multivariable created i.e. which variables were selected and added into that final model? Did all the variables in the bivariable meet the p-value cutoff you stated you used in moving to the multivariable regression? | A chi-square statistic is a measure of the difference between the observed and expected frequencies of the outcomes of a set of events or variables. Chi-square can be used to test whether two variables are related or independent from one another. It can also be used to test the goodness-of-fit between an observed distribution and a theoretical distribution of frequencies  The bivariable logistic regression is a model used to test whether a single independent variable and the outcome variable has an association. Whereas, the multivariable logistic regression is a model used to identify the significantly associated variables after introducing many independent variables at a time and a single independent variable. In this study, the variables put in the regression table meet the p-value cutoff point. |
| 06 | What was only displayed in the table was the COR and AOR with p-value only for the AOR. I think it may be good to make this much clearer. | Thank you, dear reviewer. Since the CI is more descriptive, we only indicate p-values for significantly associated variables. |
| 07 | It would have been nice to add some write up on the results from the bivariable analysis before detailing on the multivariable adjusted ones | Thank you, dear reviewer. We did it in the revised manuscript. Please kindly see the revised manuscript. |
| 08 | There is a need to rework and format your reference list for consistency as some had missing author’s name (e.g. ref. 23), more authors than the original paper (e.g. ref3), wrong citation (ref 2), typos, etc. It would also be nice to add the doi for those articles that have | Thank you, dear reviewer. We went through the entire reference and revised it based on your kind suggestion. Besides, the DOI has been added for those articles that have. Please kindly take a look at the revised manuscript. |
| 09 | Please can you explain the choice of the use of 50% for previous prevalence (p) for the sample size calculation? Can you provide the reference or provide justification for the choice | Thank you, dear reviewer. Since there was no recent similar study, we used 50%. A sentence describing this has been added in the revised manuscript. |
| 10 | There are lots of grammatical errors that may need the article to be copy edited to improve on its language structure. I have highlighted a few in the body of the article text. | Thank you, dear reviewer, for your kind suggestions and valuable help. We went through the whole document and look for grammar errors. The highlights you made were beyond crucial. Thank you once again. |
| 11 | Is it stratified simple random sampling or stratified random sampling? | Thank you, dear reviewer. It is stratified random sampling and corrected in the revised manuscript. |
| 12 | Other minor issues and comments especially for grammatical issues are as contained in my comments and highlight in the article | Thank you, dear reviewer. Corrected in the revised manuscript. |
| 13 | Suggestions and comments in the body of the article text | All the suggestions and comments on the PDF are clearly addressed. Please kindly see the revised manuscript. Thanks once again for your priceless contribution to the improvements of this manuscript. |

**Reviewer #2**

| S.N | Comments | Responses | |  |
| --- | --- | --- | --- | --- |
| 01 | Very interesting article. This is a relevant social and medical issue that must be discussed | | Thank you, dear reviewer, for your support and valuable contribution to the improvement of the manuscript. | |
| 02 | The keywords do not reflect the purpose of the article - for example, the MeSH term "Rural Health Services" could be included | | Thank you, dear reviewer. It has been included in the revised manuscript. | |
| 03 | It's necessary to revise the text. For example: - "The willingness of students to work in the rural areas was the outcome variable, while age, sex..." - Incomplete sentence | | Thank you, dear reviewer. It has been corrected in the revised manuscript. | |
| 04 | Students were asked whether they have a willingness to work in rural areas or not after graduation. Students who said “yes” when they are asked, whether they are willing to work in the rural areas after graduation or not, was considered..." – Repetition | | Thank you, dear reviewer. It has been revised, please see the revised manuscript. | |
| 05 | who hadn’t have intention..." - Wrong past tense who hadn’t have intention..." - Wrong past tense | | Thank you, dear reviewer. We did a revision, please see the revised manuscript. | |
| 06 | Besides, the multivariable logistic regression analysis revealed that being male, had an intention to continue with the profession, had a mother with no formal education..." - Wrong tense | | Thank you, dear reviewer. A revision has been made. | |
| 07 | were variables having a significant association" - Wrong tense | | Thank you, dear reviewer. We did a correction in the revised manuscript. | |
| 08 | This could be explained by parents might have a big influence on shaping and directing the attitude and behaviors of their children, including educations what to learn and work something else." - Educations what to learn? | | Thank you, dear reviewer. We did a revision, please see the revised manuscript | |
| 09 | "as compared to those students who hadn’t intended to continue with the profession that they have currently-Wrong past tense | | Thank you, dear reviewer. We did a revision based on your kind suggestion | |
| 10 | Because those students with no intention to continue with their profession might have a plan to participate..." - incomplete sentence | | Thank you, dear reviewer. “Because those students with no intention to continue with their profession might have a plan to participate in other income-generating activities and plan to change their profession due to lack of interest in the department after they engaged in”. The sentence is what we write above. | |
| 11 | This study also was found that students’ attitudes" - Also was found? - "having a mother of low academic achievement was observed to affect" --> were observed | | Thank you, dear reviewer. It has been checked and revised based on your kind recommendation. | |
| 12 | The first paragraph of the discussion was the repetition of the results. | | Thank you, dear reviewer. We did a revision and the text has been removed due to the repetition. | |
| 13 | In the conclusion, "having a good attitude towards working in rural areas" was repeated. Also "Moreover, benefits improvement is essential for students to stay in their profession without getting disappointed and planned to leave their profession." was not raised in the discussion. | | Thank you, dear reviewer. The repetition has been deleted and the last sentence has also been deleted. | |
| 14 | Table 1: > 25 ≥25 --> I think it was < 25 and ≥25 | | Thank you, dear reviewer. It was typing error and corrected in the revised manuscript. | |
| 15 | The monthly income (Average monthly pocket money) is in what currency? Dollar? | | Thank you, dear reviewer. Please accept our apologies for problem caused, it was typing error. The currency is in Ethiopian Birr (ETB). | |
| 16 | Table 2: "chat chewing" --> isn't khat chewing? | | Thank you, dear reviewer. It has been corrected. | |
| 17 | You don't need to put rows for "yes" and "no" in the table. You can just put "yes". Example: History of chat chewing, Yes 33 91.8 | | Thank you, dear reviewer. Dear reviewer, you are right it is possible to proceed as per your kind recommendation. If you don’t mind, we prefer to continue as it is to make it clear the reader and no confusion will create to the audience. | |
| 18 | English must be revised. However, I reinforce the value of the article and thematic | | Thank you, dear reviewer, for your priceless contribution you made for the improvements of the manuscript. We went through the whole document for English language and grammars. Please kindly see the revised manuscript. | |
