# Supplementary material for: Willingness to work in rural areas and associated factors among graduating health students at the University of Gondar, northwest Ethiopia, 2021
Source: PLoS One. 2022 Oct 21;17(10):e0276594. doi: 10.1371/journal.pone.0276594 (PMC9586395; doi:10.1371/journal.pone.0276594)
Supplement: Attachment — Submitted filename: Response to Reviewers.docx [file pone.0276594.s006.docx]

**August 22, 2022**

**Manuscript ID:** PONE-D-21-30311**R1 (**Willingness to work in rural areas and associated factors among graduating medical and health science students at the University of Gondar, northwest Ethiopia, 2021)

**Response to Reviewers**

First of all, we would like to thank the reviewers and the editor for their constructive comments and valuable improvements to the manuscript. We have incorporated all the suggestions and comments. The changes made during revision have been shown with tracked changes and a point-by-point response-please see below.

**Response to Reviewer(s) comments**

**Reviewer #1**

| S.N | Comments | Responses |
| --- | --- | --- |
| 01 | I believe the use of the heterogeneous group of different cadres of health personnel is not adequate for the following reasons: 1.1 WHO in its world health report 2006 (World Health Organization. (‎2006)‎. The world health report: 2006: working together for health) has clearly defined health professionals to include doctors, nurses and midwife in training hence comparison cannot be made lumping up other classes of health workers in training who ordinarily may not be heavily impacted by the issue under consideration given that their services are not always primarily needed as core areas for health provision mandate in rural areas. | Thank you, dear reviewer, for your deep review and thoughtful comment. We understand you concern and tried to address all your comments during th first revision. The mentioned document was prepared (i.e. WHO) was prepared almost 16 years back, where many things have been changed since that time including the emerging of new departments and the demand of different health professionals for the rural community. Whether the study populations are diversified not, the matter is are they willing to work in the rural area? The study was not intended to assess the primarily needed professions. Rather based on the need, we tried to assess the students’ willingness. Being diversified is a factor for the outcome variable. As far as all the mentioned professions are needed for the rural community, what is the concern to stick only one category of professionals like medicine or nursing or Midwifery? You are right, according to the WHO 2006 report, the frontline healthcare providers for the rural community are nurses, midwives, and medical doctors. However, it is not entirely mean that the other professionals/professions are not needed in rural areas. It is well known that majority of the health workers in rural areas are the abovementioned professions. Though the number matters, every profession is needed in rural areas as far as health facilities and the problem is there. Lastly, we put your concern as limitation of the study. Thank you for the understanding |
| 02 | The previous works cited and compared with your results clearly have defined their works with a specific group of class of health worker student’s usually medical students.  Given the difference in training, scope, primary job deployment (e.g. PHC workers for rural areas) and their need in rural areas amongst others, it may be difficult to lump all the classes of health workers student together. Thus given the foregoing it may be necessary to substantially redo the recruitment and focus either on health professional’s student - medical and nursing students or the other cadres who ordinarily are expected to be found in the rural areas in the PHC. Then the analysis will focus on either of the group with a new calculated sample size. | Thank you, dear reviewer. Yes you are right, previously studies use medical students only. As we try to describe above, all professions are need though the number matters. Dear reviewer, we appreciate the time you devoted to the improvement of the manuscript. However, it is not convincing for us to analyze the data for specific department only. It doesn’t affect the outcome variable, rather it will increase the quality of the study and its generalizability. Thank you for your understanding. |
| 03 | A minor edit on your reference 7 link as well as other references I have added as a comment in the Pdf | Thank you, we did it. |
|  | Reviewer #2 | |
| 01 | Congratulations to the authors for the attention given to the reviewers' comments. The quality of the article has significantly improved. | Thank you, dear reviewer, for your time and commitment for the significant improvement of the manuscript. |
