# Supplementary material for: Willingness to work in rural areas and associated factors among graduating health students at the University of Gondar, northwest Ethiopia, 2021
Source: PLoS One. 2022 Oct 21;17(10):e0276594. doi: 10.1371/journal.pone.0276594 (PMC9586395; doi:10.1371/journal.pone.0276594)
Supplement: Attachment — Submitted filename: Response to Reviewers.docx [file pone.0276594.s007.docx]

**September 20, 2022**

**Manuscript ID:** PONE-D-21-30311**R2 (**Willingness to work in rural areas and associated factors among graduating medical and health science students at the University of Gondar, northwest Ethiopia, 2021)

**Response to Reviewers**

First of all, we would like to thank the reviewers and the editor for their constructive comments and valuable improvements to the manuscript. We have incorporated all the suggestions and comments. The changes made during revision have been shown with tracked changes and a point-by-point response-please see below.

**Response to Reviewer(s) comments**

**Reviewer #1**

| S.N | Comments | Responses |
| --- | --- | --- |
| 01 | I would differ slightly concerning the relevance of the WHO 2006 report which is still one of the fundamental documents when discussing around Human resource for Health issue. Even going through the report by Feysia, Berhanu & Herbst, Christopher & Lemma, Wuleta & Soucat, Agnes. (2012). The Health Workforce in Ethiopia: Addressing the Remaining Challenges. 10.13140/RG.2.2.25472.84482 and other seminal HRH papers produced by WHO and other organization some of the issue I have raised has remained.  Going forward I think it would be best you change the term `medical and health students` to simply `health students` including in the title and every other parts of the article. For instance the title may now read “Willingness to work in rural areas and associated factors among graduating Health students at the University of Gondar, northwest Ethiopia, 2021”.  This is because fundamentally all the groups mentioned are health students. This I believe will allow the reader not to be confused given the conjoined terms is not universally used. The reader can then understand that it encompasses all students studying in one or the other of the health sciences, which I believe is what your study was about | Thank you, dear reviewer, for your kind suggestion and continuous support. Corrections have been made in the revised manuscript as per your kind recommendation. |
| 02 | In the limitation paragraph you mentioned “Besides, the study lumped all health professional together against to the WHO definition of healthcare providers for the rural community. However, all healthcare professionals…”  I am thinking would it not be better specifying students studying one of the health courses since your study participants are currently graduating students…. | Thank you, dear reviewer. Revision has been made in the revised manuscript. Please kindly see the revised manuscript. Once again thank you for your time! We highly appreciate the reviewer for their priceless support for the significant improvement of this manuscript. |
